# Sustainable Design and Environmental Effects of π-Conjugated Thiophene Surfactants for Optoelectronic Applications

**DOI:** 10.3390/ma18184349

**Published:** 2025-09-17

**Authors:** Catalina Stoica, Hisham Idriss, Justin Z. Lian, Julie-Lisa Malaval, Anca-Maria Patrascu, Alina Roxana Banciu, Stefano Cucurachi, Sébastien Richeter, Sébastien Clément, Mihai Nita-Lazar

**Affiliations:** 1 National Research and Development Institute for Industrial Ecology—ECOIND, 57-73 Drumul Podu Dambovitei, District 6, 060652 Bucharest, Romania; catalina.stoica@incdecoind.ro (C.S.); anca.harabagiu@ecoind.ro (A.-M.P.); alina.banciu@incdecoind.ro (A.R.B.); 2 Institut Charles Gerhardt Montpellier (ICGM), University of Montpellier, Centre National de la Recherche Scientifique (CNRS), École Nationale Supérieure de Chimie de Montpellier (ENSCM), Balard Recherche, Pôle Chimie, ICGM UMR 5253, 1919 Rte de Mende, 34293 Montpellier Cedex 5, France; hisham.idrissk@gmail.com (H.I.); julie-lisa.malaval@etu.umontpellier.fr (J.-L.M.); sebastien.richeter@umontpellier.fr (S.R.); sebastien.clement1@umontpellier.fr (S.C.); 3 Leiden University, Institute of Environmental Sciences—Industrial Ecology, Van Steenisgebouw, Einsteinweg 2, 2333 CC Leiden, The Netherlands; z.lian@cml.leidenuniv.nl (J.Z.L.); s.cucurachi@cml.leidenuniv.nl (S.C.)

**Keywords:** non-pathogenic bacteria adhesion, optoelectronic surfactants, amphiphilic optoelectronic molecules, oligothiophenes, ProDOT, organic materials for sensors and devices

## Abstract

Electronic waste is becoming a growing global pollution issue due to short device lifespans and insufficient safe disposal methods. Hazardous metals like arsenic and mercury from electronic waste harm both the environment and human health. Recycling processes remain underdeveloped, requiring new eco-friendly solutions. This paper reports on the synthesis and properties of the cationic surfactants ammonium terthiophene (**CTT**) and 3,4-propylene-dioxythiophene (**C-ProDOT**), which may have potential use in organic electronics. Ecotoxicological tests showed no significant long-term toxicity and medium-to-high biodegradability, which are keys for environmental protection. These surfactants also displayed selective bacterial adhesion, making them candidates for bionic devices. Life cycle assessment revealed higher energy use and ecotoxicity for **C-ProDOT** than **CTT**, underscoring the need for sustainable chemical design.

## 1. Introduction

Population and economic growth have triggered a surge in the production and use of electronic devices. Worldwide “electronification” and digital transformation have profoundly reformed human society’s approach to living, working, learning and communicating. Rapid progress in the electronics industry and technology, as well as shorter product life cycles, have led to intensified global mining for rare elements, from almost 50,000 metric tons in 1985 to 300,000 metric tons in 2023 according to the US Geological Survey, 2023 [1] and the Bureau of Mines Minerals Yearbook (1985–1993) [2]. Unfortunately, the massive production of electronics has resulted in a significant accumulation of electronic waste (e-waste), largely due to inefficient waste management and limited recycling infrastructure, triggering environmental degradation and health hazards. Generally, e-waste encompasses a broad range of electronic devices such as laptops, smartphones, televisions, household appliances and other gadgets, devices which guarantee a comfortable existence for people [3,4]. According to the Global E-waste Monitor [4], an estimated 62 billion kilograms of e-waste was produced globally by 2022, which averages to less than 8 kg per person. Unfortunately, around 13.8 billion kg of all electronics produced and sold worldwide have been recycled or reused, while the remaining 75% has become e-waste [4,5]. Additionally, global e-waste generation is projected to increase by 25.7%, reaching 74.7 million metric tons by 2030 [6].

Globally, Asia produces the highest volume of e-waste, approximatively 25 million metric tons, followed by North and South America with around 13 million metric tons (the United States alone produces around 7 million metric tons), Europe with about 12 million metric tons, Africa with around 2.9 million metric tons and Oceania with around 0.7 million metric tons annually [7].

Consequences of improper electronic waste management were associated with discharges of toxic chemicals including metals, dioxins, polychlorinated biphenyl or polycyclic aromatic hydrocarbons into the environment, affecting the soil, water, air and consequently, human health [8,9]. Wastewater treatment plants (WWTPs) have been used to remove a broad spectrum of pollutants originating from both domestic and industrial sources, and they rely on two major steps: biological and physico-chemical steps. Applications of microorganisms for heavy metal removal have gained attention due to their versatile adaptation to extreme environmental conditions, enabled by rapid bacterial mutations and evolutionary changes. Despite the fact that bacteria have been employed to remove heavy metals [10,11], their efficiency has been marginally successful. The percentage distribution of individual heavy metals in WWTP effluent and activated sludge streams revealed that over 70% of Mn and Cu were retained in the sludge, whereas 45–63% of Cd, Cr, Pb, Fe, Ni, Zn remained in the effluent [12,13], potentially allowing for their discharge into the environment and generating significant negative impacts on human health and environmental ecosystems [14].

In recent decades, the necessity of finding alternative eco-friendly compounds with desirable electronic properties that can be easily biodegraded has become increasingly clear. In this context, conjugated oligomers and polymer-based surfactants have been promising materials that combine their conjugated backbone electronic properties with the amphiphilic nature of surfactants. Their ability to conduct electricity makes them powerful advanced functional materials to be used in modern organic optoelectronic devices, including electrochromic devices, organic semiconductors and sensors [15,16,17,18,19,20]. In particular, oligo/polythiophene-based surfactants have notably demonstrated enhanced planarity and π-delocalization, exhibiting excellent optoelectronic properties, making them candidates for high performance optoelectronics [21].

A particular area of interest is their supramolecular electronic properties, which involve nanoscale structural features formed from the self-assembly of molecular components [22]. Conducting films based on the gas–water interface have shown great potential as a platform to study the self-assembly of π-conjugated oligomers and polymers [23,24,25]. In this platform, the “substrate” (water) is unique within the architecture of a soap film, consisting of a quasi-2D semipermeable membrane stretched by surface tension and stabilized by self-assembled surfactant monolayers that sandwich the aqueous phase [24,26]. Thiophene-based surfactants could be used in biosensing applications due to their combination of conjugated electronic properties, amphiphilicity and biocompatibility, particularly their ability to interact with biological molecules and provide optical or electrochemical signals [27,28,29].

The present work presents the design of new cationic terthiophene (**CTT**) and 3,4-propylene-dioxythiophene (**C-ProDOT**) surfactants as candidates for optoelectronic applications.

These surfactants are intended to address e-waste pollution and thus their entire chemical synthesis process was analyzed through life cycle assessment (LCA). In addition, their eco-friendly properties were tested through biodegradability and ecotoxicological impact tests conducted on a non-pathogenic bacterial model. The cellular adhesion and toxic properties of **CTT** and **C-ProDOT** surfactants, essential for optoelectronic device cellular signal transduction, were assessed in the presence of a bacterial model.

Overall, the newly designed **CTT** and **C-ProDOT** surfactants exhibited bacterial adhesion and no significant long-term toxicity to bacterial cells. These compounds could serve as a backbone supporting various applications, including non-toxic organic semiconductors and advanced organic sensors [30].

## 2. Materials and Methods

### 2.1. Synthesis of the Amphiphilic Thiophenes

**Scheme 1:**
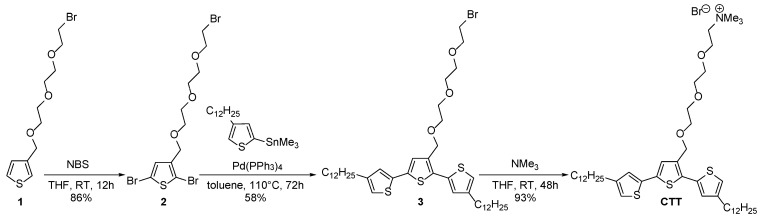
Synthesis of cationic-based terthiophene surfactant **CTT**. To enhance the hydrophilic properties, a ProDOT molecule with two oligoether chains (**6**) was selected as the central core and was synthesized through a two-step process: (i) alkylation of dihydroxy-ProDOT **4**, followed by (ii) dibromination of compound **5** using NBS. A Stille cross-coupling reaction was then performed between (2-trimethylstannyl)-4-dodecylthiophene and **6,** affording **7** in 60% yield. Finally, **7** was converted in 95% yield into the corresponding ammonium-based ProDOT **C-ProDOT** by reaction with trimethylamine (Scheme 2). Both **CTT** and **C-ProDOT** have been thoroughly characterized using NMR spectroscopy and high-resolution mass spectrometry (See the Supplementary Materials).

**Scheme 2:**
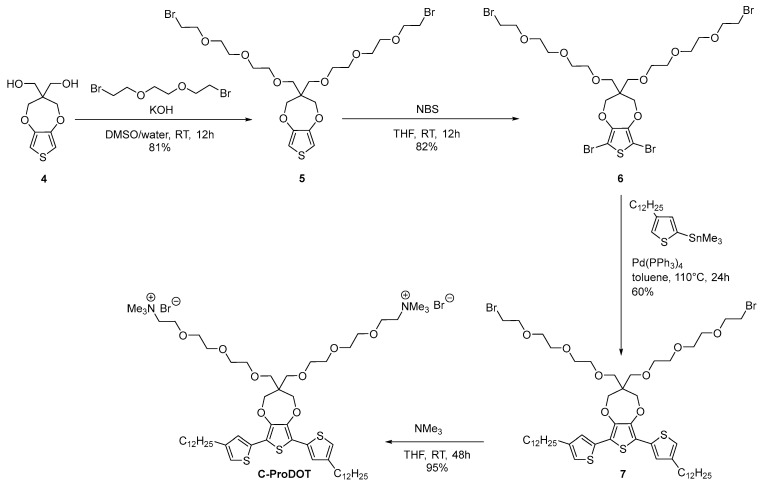
Synthesis of **C-ProDOT**-based ionic surfactant.

**Materials**. Reactions were performed under nitrogen atmosphere. All anhydrous solvents and reagents were purchased from commercial suppliers: THF (Merck, Darmstadt, Germany, 99.9%), DMSO (Thermo Fisher Scientific, Waltham, MA, USA, 99.7%), toluene (Thermo Fisher Scientific, Waltham, MA, USA 99.85%), N-bromosuccinimide (Merck, Darmstadt, Germany 99%), trimethylamine in solution in THF (2 M) (Merck, Darmstadt, Germany). Pd(PPh_3_)_4_, (2-trimethylstannyl)-4-dodecylthiophene, 1-Bromo-2-(2-(2-(2-bromoethoxy)ethoxy)ethoxy)ethane and compounds **1** and **4** (Schemes 1 and 2) were prepared according to the literature [31,32,33,34,35]. Thin-layer chromatography (TLC) was performed on Merck DC Kieselgel 60 F-254 aluminum plates, with spot visualization under a UV lamp. Preparative purifications were conducted using silica gel column chromatography (Merck 40−60 μm), while flash chromatography was executed with Biotage^®^ Isolera™ Systems.

**Synthesis of 2**. In a two-neck round bottom flask, **1** (1.00 g, 3.25 mmol) was dissolved in 15 mL of anhydrous THF. Gradual addition of N-bromosuccinimide (1.27 g, 7.15 mmol, 2.2 equivalents) followed. The reaction mixture was stirred at room temperature for 12 h. Afterward, the solution was concentrated under reduced pressure. 20 mL of water was added to the mixture. The organic layer was extracted with diethyl ether (3 × 20 mL), dried with anhydrous MgSO_4_ and filtered. The solvent was eliminated under reduced pressure. The obtained residue was purified via column chromatography on silica gel with a *n*-hexane/ethyl acetate mixture (8:2, v/v) as the eluent, yielding compound **2** as a yellow oil (1.29 g, 86%). ^1^H NMR (600 MHz, CDCl_3_) δ 7.23 (s, 1H), 4.67 (s, 2H), 4.05 (t, ^3^*J*_H-H_ = 6.3 Hz, 2H), 3.93–3.88 (m, 6H), 3.87–3.84 (m, 2H), 3.71 (t, ^3^*J*_H-H_ = 6.3 Hz, 2H) ppm. ^13^C{^1^H} NMR (101 MHz, CDCl_3_) δ 138.2, 129.9, 110.2, 108.9, 70.22, 69.6, 69.55, 69.54, 68.6, 65.9, 29.3 ppm. HRMS (ESI^+^): *m/z* calc for C_11_H_15_Br_3_O_3_S [M + H]^+^: 464.8365 Da, found: 464.8372 Da.

**Synthesis of 3**. In a two-neck round-bottom flask, compound **2** (0.60 g, 1.29 mmol), (2-trimethylstannyl)-4-dodecylthiophene (1.08 g, 2.58 mmol, 2 equivalents) and the catalyst Pd(PPh_3_)_4_ (0.15 g, 0.13 mmol, 0.10 equivalents) were dissolved in 10 mL of toluene. The mixture was stirred at 110 °C for three days, after which it was concentrated under reduced pressure. Following concentration, 20 mL of water was added to the reaction mixture. The organic layer was extracted with diethyl ether (3 × 20 mL), dried over anhydrous MgSO_4_ and filtered. The solvent was removed using a rotary evaporator. The resulting residue was purified by column chromatography on silica gel using a *n*-hexane/ethyl acetate mixture (7:3, v/v) as the eluent, resulting in the recovery of compound **3** as a brown solid (0.60 g, 58%). ^1^H NMR (400 MHz, CDCl_3_) δ 7.16 (s, 1H), 7.01 (d, ^3^*J*_H-H_ = 1.4 Hz, 1H), 7.00 (d, ^3^*J*_H-H_ = 1.3 Hz, 1H), 6.91 (d, ^3^*J*_H-H_ = 1.1 Hz, 1H), 6.80 (d, ^3^*J*_H-H_ = 1.0 Hz, 1H), 4.60 (s, 2H), 3.80 (t, ^3^*J*_H-H_ = 6.3 Hz, 2H), 3.72–3.68 (m, 4H), 3.67 (s, 4H), 3.45 (t, ^3^*J*_H-H_ = 6.3 Hz, 2H), 2.63–2.55 (m, 4H), 1.69–1.58 (m, 4H), 1.36–1.28 (m, 8H), 1.27 (s, 28H), 0.89 (t, ^3^*J*_H-H_ = 6.8 Hz, 6H) ppm. ^13^C{^1^H} NMR (126 MHz, CDCl_3_) δ 144.2, 144.1, 136.6, 135.7, 135.4, 134.6, 133.1, 127.8, 126.1, 125.1, 120.8, 119.2, 71.3, 70.7, 70.6, 69.5, 67.0, 32.0, 30.7, 30.5, 30.4 30.4, 29.7, 29.7, 29.7, 29.5, 29.4, 29.4, 22.8, 14.2 ppm. HRMS (ESI^+^): *m/z* calc for C_43_H_69_BrO_3_S_3_ [M + NH_4_]^+^: 826.3930 Da, found: 826.3921 Da.

**Synthesis of CTT**. In a Schlenk tube, compound **3** (0.10 g, 0.12 mmol) was dissolved in 3 mL of dry THF. Subsequently, trimethylamine (4 mL, 2 M in THF) was added. The mixture was stirred at room temperature for two days. After the stirring period, the solution was concentrated under reduced pressure. Following concentration, 10 mL of water was added, and the organic layers were extracted with diethyl ether (2 × 10 mL) and dichloromethane (2 × 10 mL). The aqueous layer was then lyophilized, yielding **CTT** (0.10 g, 95%). ^1^H NMR (400 MHz, CDCl_3_) δ 7.11 (s, 1H), 7.00–6.98 (m, 2H), 6.93 (d, ^3^*J*_H-H_ = 0.8 Hz, 1H), 6.81 (d, ^3^*J*_H-H_ = 0.8 Hz, 1H), 4.52 (s, 2H), 3.93–3.88 (m, 2H), 3.88–3.85 (m, 2H), 3.67–3.62 (m, 8H), 3.30 (s, 1H), 3.22 (s, 8H), 2.62–2.53 (m, 4H), 1.61–1.57 (m, 4H), 1.41 (s, 1H) 1.31 (s, 8H), 1.25 (s, 28H), 0.87 (t, ^3^*J*_H-H_ = 6.8 Hz, 6H) ppm. ^13^C{^1^H} NMR (101 MHz, CDCl_3_) δ 144.5, 144.2, 136.0, 135.7, 135.1, 134.2, 133.4, 127.9, 126.1, 125.4, 121.1, 119.5, 70.3, 70.2, 70.0, 69.5, 66.8, 65.5, 65.1, 54.6, 31.9, 30.5, 30.4, 30.4, 29.7, 29.6, 29.5, 29.4, 22.7, 14.1 ppm. HRMS (ESI+): *m/z* calc for C_46_H_78_NO_3_S_3_ [M]^+^: 788.5138 Da, found: 788.5147 Da.

**Synthesis of 5**. In a two-neck round-bottom flask, a mixture containing 3,3-di(2-[2-(2-bromoethoxy)ethoxy]ethoxy]methyl)-3,4-dihydro-2H-thieno [3,4-b][1,4]dioxepine (**4**) (0.90 g, 4.16 mmol), *n*-tetrabutylammonium bromide (0.20 g, 0.62 mmol, 0.15 equivalents) and potassium hydroxide (1.56 g, 27.87 mmol, 6.7 equivalents) was prepared by dissolving in 4.5 mL of water. Subsequently, a solution of 1-bromo-2-(2-(2-(2-bromoethoxy)ethoxy)ethoxy)ethane (7.99 g, 24.96 mmol, 6 equivalents) in 10 mL of DMSO was introduced dropwise into the reaction mixture. The solution was stirred for 12 h at room temperature. Following this period, an additional 10 mL of water was introduced to the mixture. The organic phase was extracted with diethyl ether (3 × 20 mL). The combined organic extracts were then dried using anhydrous MgSO_4_, filtered and concentrated under reduced pressure. The resulting residue was subject to purification via silica gel column chromatography, employing a *n*-hexane/ethyl acetate mixture (1:1, v/v) as the eluent, resulting in the isolation of compound **5** as a light yellow oil (2.03 g, 81%). ^1^H NMR (400 MHz, CDCl_3_) δ 6.43 (s, 2H), 4.00 (s, 4H), 3.78 (t, ^3^*J*_H-H_ = 6.3 Hz, 4H), 3.65–3.56 (m, 16H), 3.55 (s, 4H), 3.44 (t, ^3^*J*_H-H_ = 6.3 Hz, 4H) ppm.

**Synthesis of 6**. In a two-neck round-bottom flask, **5** (0.80 g, 1.32 mmol) was dissolved in anhydrous THF (20 mL) and cooled to 0 °C. The solution underwent degassing through three cycles of freeze–pump–thaw. N-bromosuccinimide (0.52 g, 2.90 mmol, 2.2 eq) was then added portionwise and the mixture was stirred at ambient temperature overnight. After this, the solution was concentrated under vacuum. Following concentration, 10 mL of water was added and the organic phase was extracted with diethyl ether (3 × 20 mL). The organic extracts were dried with anhydrous MgSO_4_, filtered and again concentrated under reduced pressure. The resulting residue was subject to column chromatography on silica gel using a mixture of *n*-hexane/ethyl acetate (8:2, v/v) as eluent, leading to **6** as a light yellow oil in 82% yield (0.83 g). ^1^H NMR (400 MHz, CDCl_3_) δ 4.09 (s, 4H), 3.81 (t, ^3^*J*_H-H_ = 6.3 Hz, 4H), 3.69–3.65 (m, 8H), 3.64–3.58 (m, 8H), 3.58 (s, 4H), 3.48 (t, ^3^*J*_H-H_ = 6.3 Hz, 4H) ppm. ^13^C{^1^H} NMR (101 MHz, CDCl_3_) δ 147.1, 91.2, 74.0, 71.2, 71.1, 70.6, 70.6, 70.4, 69.8, 47.9, 30.6 ppm. HRMS (ESI^+^): *m/z* calc for C_21_H_32_Br_4_O_8_S [M + H]^+^: 760.8624 Da, found: 760.8627 Da.

**Synthesis of 7**. To a two-neck round-bottom flask, **6** (0.50 g, 0.65 mmol), (2-trimethylstannyl)-4-dodecylthiophene (0.54 g, 1.31 mmol, 2 eq), tetrakis(triphenylphosphine)palladium (0) (0.08 g, 0.26 mmol, 0.1 eq) were added together in 10 mL of toluene. The mixture was then stirred at 110 °C for 24 h. After the reaction period, the solution was concentrated under vacuum. Subsequently, 10 mL of water was added and the organic phase was extracted using diethyl ether (three times with 20 mL each). The combined organic extracts were dried over anhydrous MgSO_4_ and filtered, and the solvent was evaporated under reduced pressure. The remaining residue was purified using silica gel column chromatography, employing a mixture of *n*-hexane and ethyl acetate mixture (7:3, v/v) as the eluent, leading to **7** as a yellow oil in 60% yield (0.44 g). ^1^H NMR (400 MHz, CDCl_3_) δ 7.03 (d, ^3^*J*_H-H_ = 1.1 Hz, 2H), 6.82 (s, 2H), 4.18 (s, 4H), 3.79 (t, ^3^*J*_H-H_ = 6.3 Hz, 4H), 3.66 (s, 12H), 3.64 (d, ^3^*J*_H-H_ = 1.4 Hz, 8H), 3.44 (t, ^3^*J*_H-H_ = 6.3 Hz, 4H), 2.57 (t, ^3^*J*_H-H_ = 7.6 Hz, 4H), 1.61 (quint, ^3^*J*_H-H_ = 7.1 Hz, 4H), 1.37–1.29 (m, 8H), 1.27 (s, 28H), 0.88 (t, ^3^*J*_H-H_ = 6.8 Hz, 6H) ppm. ^13^C{^1^H} NMR (126 MHz, CDCl_3_) δ 145.1, 143.1, 134.2, 124.2, 119.3, 114.2, 73.8, 71.3, 71.2, 70.7, 70.6, 70.5, 70.2, 48.0, 32.0, 30.5, 30.5, 30.4, 29.8, 29.7, 29.7, 29.6, 29.4, 29.4, 22.8, 14.2 ppm. HRMS (ESI^+^): *m/z* calc for C_53_H_86_Br_2_O_8_S_3_ [M + H]^+^: 1105.3934 Da, found: 1105.3924 Da.

**Synthesis of C-ProDOT**. In an oven-dried Schlenk flask, compound **7** (75 mg, 0.07 mmol) was dissolved in anhydrous THF (3 mL). A 2.0 M solution of trimethylamine in THF (3 mL) was then added to the reaction mixture. The resulting solution was stirred at room temperature for 3 days. After the reaction was complete, the solvent was removed under vacuum. Water (10 mL) was then added and the product was extracted with diethyl ether (2 × 10 mL) and dichloromethane (2 × 10 mL). The aqueous phase was then lyophilized, affording the desired ionic surfactant **C-ProDOT** in 95% yield (0.07 g). ^1^H NMR (400 MHz, CDCl_3_) δ 6.99 (d, ^3^*J*_H-H_ = 1.3 Hz, 2H), 6.82 (d, ^3^*J*_H-H_ = 1.1 Hz, 2H), 4.12 (s, 4H) 4.01–3.95 (m, 4H), 3.92–3.87 (m, 4H), 3.70–3.60 (m, 4H), 3.64–3.61 (m, 6H), 3.60 (s, 8H), 3.45 (s, 18H), 2.55 (t, ^3^*J*_H-H_ = 7.6 Hz, 4H), 1.58 (quint, ^3^*J*_H-H_ = 7.2 Hz, 4H), 1.33–1.28 (m, 10H), 1.24 (s, 28H), 0.86 (t, ^3^*J*_H-H_ = 6.8 Hz, 6H) ppm. ^13^C{^1^H} NMR (101 MHz, CDCl_3_) δ 145.0, 143.2, 133.9, 124.3, 119.5, 114.3, 73.8, 71.3, 70.5, 70.4, 70.3, 70.0, 65.5, 65.2, 54.6, 51.9, 48.1, 34.1, 31.9, 30.5, 30.4, 29.7, 29.7, 29.6, 29.5, 29.4, 22.7, 22.3, 14.1, 14.1 ppm. HRMS (ESI+): *m/z* calc for C_59_H_104_N_2_O_8_S_3_ [M]^2+^: 532.3472 Da, found: 532.3471 Da.

### 2.2. Characterization Methods

Nuclear Magnetic Resonance (NMR) spectra, including ^1^H and ^13^C{^1^H} NMR, were obtained using Bruker Avance III HD 400 MHz and 500 MHz (Bruker, Billerica, MA, USA) spectrometers operating at 298 K. The deuterated solvents, CDCl_3_ (Sigma-Aldrich, Saint-Louis, MO, USA, 99.8%) and DMSO-*d*6 (Avantor, Radnor Township, PA, USA, >99.0%), were utilized as received. The ^1^H and ^13^C{^1^H} NMR spectra were calibrated against the residual non-deuterated solvent, which served as an internal standard for chemical shifts, expressed in ppm (δ). The following abbreviations were used in the NMR spectra: s for singlet, d for doublet, t for triplet and m for multiplet. High Resolution Mass Spectrometry (HRMS) was conducted on a Bruker MicroTof QII instrument (Bruker, Billerica, MA, USA) operating in both positive and negative modes (ESI).

### 2.3. Optical Methods

UV–visible absorption spectra were obtained using a JASCO V-750 UV-Visible-NIR spectrophotometer (JASCO France, Lisses, France) in 10 mm quartz cells (Hellma, Müllheim, Germany), with measurements conducted in THF and water. Molar extinction coefficients (ε) were calculated by preparing surfactant solutions at various concentrations in both solvents, ensuring that the concentrations fell within the linear range of the Beer–Lambert law (absorbance approximately 0.2–0.8). Emission spectra were collected at 298 K using a fluorescence spectrophotometer (FLS920, Edinburgh Instrument, Livingston, UK), which featured a calibrated photomultiplier housed in an air-cooled Peltier unit (R928P, Hamamatsu, Hamamatsu City, Shizuoka Pref., Japan). A 450 W continuous xenon arc lamp served as the excitation source for steady-state photoluminescence measurements conducted in a 10 mm quartz cell (Hellma, Müllheim, Germany). The quantum yields of the samples were assessed using an integrating sphere (120 mm diameter) from Edinburgh Instruments in air.

### 2.4. Dynamic Light Scattering (DLS)

Dynamic Light Scattering (DLS) measurements were conducted with a Malvern Zetasizer Nano-ZS (Malvern Instruments Ltd., Worcestershire, UK) in aqueous solutions. The critical micellar concentration (CMC) values were determined through the preparation of various surfactant solutions in water at different concentrations, followed by scattered light intensity measurements.

### 2.5. Biodegradation Potential

The biodegradation potential of the oligothiophenes-based surfactants was estimated based on their biodegradability index (Table 1) [36,37]. The biochemical oxygen demand at 5 days (BOD_5_) was quantified following SR ISO 5815–1:2020 [38] standard method. Briefly, the surfactant samples were incubated for 5 days in the dark at 20 °C in the presence of microbiologically seeded dilution water. BOD_5_ (mg O_2_/L) was calculated based on the variation in oxygen concentration from day 0 to day 5 measured by an electrochemical method.

The chemical oxygen demand (COD) was detected following the SR ISO 15705:2022 [39] standard method, where the surfactants were chemically oxidized using 5 mL of potassium dichromate in strong sulfuric acid solution.

The remaining dichromate concentration was measured, after 2 h of incubation, by titration with ammonium iron (II) sulfate using ferroin as the indicator. The COD results were reported as mg O_2_/L.

### 2.6. Surfactant Detection and Quantification

The amounts of cationic surfactants were detected according to Annex IIC of European Commission (EC) Regulation 648 (2004) [40].

The cationic surfactants interacted with disulfine blue, forming an extractable chloroform colored complex in acidic medium. The intensity of the colored complex was directly proportional to surfactant concentration and was spectrophotometrically detected using a UV–visible absorption spectrophotometer (Shimadzu, Kyoto, Japan).

### 2.7. Bacterial Respiration Rate

Bacterial strains from activated sludge from a municipal WWTP were incubated with 100 mg/L solution of oligothiophene-based surfactants under continuous stirring (Oxitop, WTW, Weilheim, Germany) for up to 1.5 h at 20 ± 2 °C. The bacterial oxygen consumption, both in the presence and absence of oligothiophene-based surfactants, was monitored using the multiparameter (Orion Star A329, Thermo Scientific, Bremen, Germany), following SR EN ISO 8192:2008 [41].

### 2.8. Bacterial Growth

Bacterial growth tests were performed on two Gram-negative bacteria, *Escherichia coli* (*E. coli*) (NCTC 12241) and *Pseudomonas putida* (*P. putida*) (ATCC 17514), and one Gram-positive bacterial strain, *Lactobacillus acidophilus* (*L. acidophilus*) (ATCC 4356). All bacterial strains were non-pathogenic and were obtained from American Type Culture Collection (ATCC, Manassas, VA, USA).

Each bacterial strain was initially cultured for 24 h at 37 °C on specific solid growth media: *E. coli* on chromocult coliform agar (Biomaxima, Lublin, Poland), *P. putida* on pseudomonas agar base (Oxoid Limited, Basingstoke, Hampshire, UK) and *L. acidophilus* on Rogosa agar (Merck Millipore, Darmstadt, Germany). A single pure colony of each bacterial strain was then transferred to 10 mL of Lauryl tryptose broth (for *E. coli* and *P. putida*) (Scharlau, Barcelona, Spain) or 10 mL MRS broth (for *L. acidophilus*) (VWR Chemicals, Leuven, Belgium) and incubated for 24 h at 37 °C, under gentle rotation at 130 rpm, in the presence or absence of 100 mg/L oligothiophene-based surfactants. The effect of the surfactants on bacterial cell growth was assessed by measuring the optical density at 600 nm (OD_600nm_) using a UV–visible absorption spectrophotometer (VWR International, Radnor, PA, USA).

### 2.9. Adhesion of Oligothiophene-Based Surfactants to Bacterial Surface

A bacterial-coated polystyrene ELISA plate was subjected to incubation with or without oligothiophene-based surfactants and its adhesion percentage was calculated based on its initial concentration and final supernatant concentration. Briefly, the ELISA plate was coated in the presence of 0.2 mL from each bacterial strain, grown to a bacterial growth density of 1 OD_600nm_ in Lauryl tryptose broth, for 1 h at room temperature. ELISA control plates were coated only in the presence of Lauryl tryptose broth. Three successive washes with phosphate-buffered saline (PBS) were performed, then 0.1 mL of 100 mg/L oligothiophene-based surfactants was added, followed by 1 h incubation at room temperature. The initial and final concentrations (unbound oligothiophene-based surfactants from the supernatant fraction after 1 h incubation) of oligothiophene-based surfactants were used to calculate their adhesion percentage.

### 2.10. Life Cycle Assessment (LCA)

LCA of the environmental impacts associated with the synthesis of **CTT** and **C-ProDOT** surfactants was conducted by following the guidelines of ISO 14040:2006 [42] and ISO 14044:2006 [43]. The system boundary of this study was defined as cradle-to-gate, with the functional unit being the synthesis of 1 g of **CTT** and **C-ProDOT** surfactants in the European region. The LCA studies used The Activity Browser (v.2.10.1) as a software tool and Ecoinvent 3.9 as a database [44], applying the EF family method for environmental impact assessment [45,46]. Life cycle inventory (LCI) data was gathered from the ICGM laboratory at the University of Montpellier.

Our data collection strategy involved direct on-site monitoring at the research laboratory where the syntheses of **CTT** and **C-ProDOT** surfactants were carried out. Detailed material consumption for each step in the synthetic pathways was recorded during the experiments. Energy usage was monitored using a power meter to track the energy consumption of equipment, including heating plates, stirrers and various other devices used throughout the duration of the reactions. Researchers and technical experts from the laboratory were consulted to verify the accuracy of the experimental data and to provide additional insight into the synthesis procedures. Stoichiometric calculations were applied for chemicals not found in the Ecoinvent database to derive their environmental impacts based on similar existing entries. The data was compiled into a life cycle inventory. Contribution analysis was performed following the LCA to identify the most impactful stages of the synthesis processes for **CTT** and **C-ProDOT** surfactants. This analysis highlighted the key contributors to the environmental burdens, such as energy-intensive steps or specific chemicals, enabling targeted recommendations for potential improvements to reduce environmental impacts.

## 3. Results and Discussion

### 3.1. Synthesis of Cationic-Based Amphiphilic Terthiophene

The cationic-based terthiophene surfactant **CTT** was prepared in three steps from compound **1** (Scheme 1). First, dibromination of precursor **1** with N-bromosuccinimide (NBS) afforded compound **2** in 86% yield. Compound **3** was then prepared via a Stille cross-coupling reaction and isolated after purification by silica gel column chromatography, with a yield of 58%. Finally, conversion towards the cationic terthiophene **CTT** involved the reaction of compound **3** with trimethylamine in THF at room temperature.

### 3.2. Aggregation Behavior

The critical micelle concentration (CMC) of these cationic oligothiophenes was determined using DLS. To determine this value, DLS was performed on aqueous solutions of **CTT** and **C-ProDOT** at a broad range of concentrations (Figures S22 and S23 in the Supplementary Materials). A plot of scattered light intensity against concentration was created. A sharp increase in scattered light is associated with micelle formation [47,48], indicating the CMC. The CMCs of **CTT** and **C-ProDOT** were found to be 0.22 and 0.32 mM in water, respectively. The obtained values are highly consistent with the previous reported data for neutral terthiophene- and ProDOT-based surfactants [25], but lower, for example, than those of the commercial surfactants sodium dodecyl sulfate (SDS, 8.0–8.2 mM) and cetyltrimethylammonium bromide (CTAB), which are both used in organic electronics [49,50].

### 3.3. Optical Properties

The optical behavior of oligothiophenes is influenced by both their internal chain conformation and the way their chains aggregate with each other. To explore this, the photophysical properties of the amphiphilic terthiophenes **CTT** and **C-ProDOT** were studied in THF and in water (Table 2). In THF, **CTT** exhibits a broad absorption band centered at 354 nm, attributed to a π-π* transition, which corresponds fairly well with the absorption maximum wavelength of terthiophene itself (355 nm) [51]. In comparison, the UV–visible absorption of **C-ProDOT** showed an increase in the absorption maximum wavelength as well as a vibronic structure due to a more rigid structure and the stronger donating effect of ProDOT group [25]. Upon transitioning to water, a bathochromic shift in the absorption maxima was observed, possibly indicating a higher conjugated terthiophene backbone in an aqueous environment.

**Figure 1:**
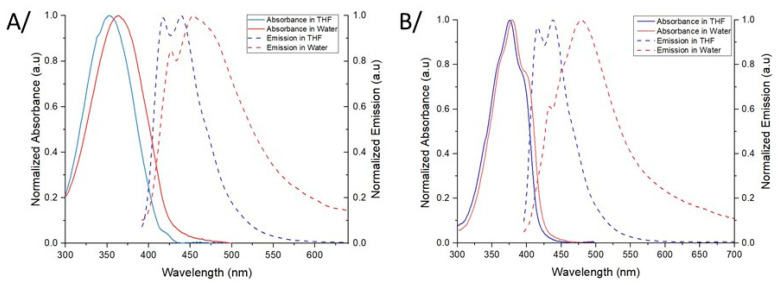
UV–visible absorption and emission spectra of (**A**)/**CTT** in water (11.40 µM, λ_exc_ = 365 nm) and in THF (11.90 µM, λ_exc_ = 355 nm) and (**B**)/**C-ProDOT** in water (10.70 µM, λ_exc_ = 380 nm) and in THF (5.63 µM, λ_exc_ = 375 nm).

A comparable trend was noted regarding their emission properties. Indeed, when the solvent polarity was increased through the transition from THF to water, **CTT** and **C-ProDOT** exhibited bathochromic shifts in their emission wavelengths of approximately 15 and 40 nm, respectively (Figure 1). This observation implies that, in an excited state, these compounds likely assume a more planar conformation. The resulting red-shift was also associated with a decrease in vibronic structure and a broadening of the emission band. Fluorescence self-quenching was observed in water, indicated by a decrease in fluorescence quantum yield. This suggests that electronic coupling occurred between self-assembled chromophores, likely due to π-stacking interactions.

### 3.4. Biodegradation Potential of Oligothiophene-Based Surfactants

Biodegradability studies were performed on **CTT** and **C-ProDOT** surfactants by analyzing their BOD_5_/COD ratio (Table 1). Their BOD_5_/COD ratio values indicated a moderate biodegradation potential for both surfactants tested (Table 3).

A moderate biodegradability rate of 0.32 BOD_5_/COD for the compounds **CTT** and **C-ProDOT** suggests that they could be processed and biodegraded by microbial communities from municipal WWTPs. The primary treatment step of industrial or domestic wastewater management involves biological processes, during which a bacterial consortium derived from activated sludge biodegrades most pollutants, including surfactants.

Resistance to surfactants, specifically quaternary ammonium compounds, is crucial for microorganisms to overcome toxicity and achieve effective removal in biological processes. Defense mechanisms include adjusting the outer membrane and porins to prevent quaternary ammonium compounds from entering the cells, utilizing efflux pumps to expel surfactants from within the cells and biodegrading the surfactants [52,53].

Surfactants’ biodegradation potential and their environmental impact were correlated with microbial respiration rates. A consortium of bacteria from activated sludge from a municipal WWTP was exposed to 100 mg/L solution of CTT and C-ProDOT, respectively.

**Figure 2:**
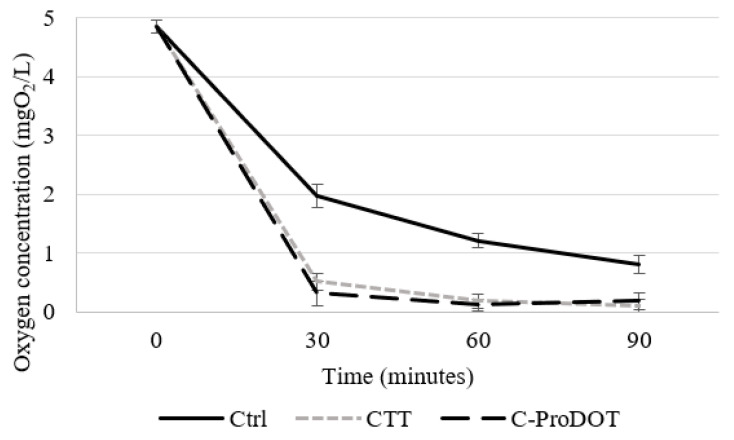
The effect of **CTT** and **C-ProDOT** compounds on microbial oxygen uptake. Control samples (Ctrl) were bacteria incubated without oligothiophene-based surfactants. All results are based on three independent experiments.

The effects of **CTT** and **C-ProDOT** surfactants on the respiration rate of the bacterial consortium exhibited a similar pattern despite differences in their chemical structures (Figure 2). Higher oxygen consumption occurred within the first 30 min of incubation with both compounds compared to the control sample (Ctrl) (bacterial consortia incubated in the absence of surfactants), leaving approximatively 38% O_2_ unused in the control sample.

**Figure 3:**
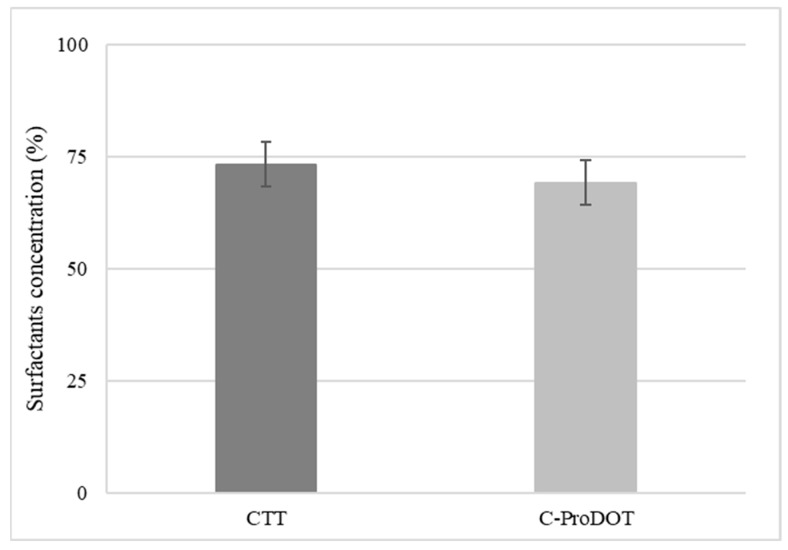
Quantification of surfactants (**CTT** and **C-ProDOT**) after 90 min of incubation. All results are based on three independent experiments.

The respiration rate was associated with the potential biodegradation of these compounds as a result of the overall metabolic processes of the bacterial consortium. The quantities of **CTT** and **C-ProDOT** were spectrometrically quantified during the biodegradation tests, both in the presence or the absence of the bacterial consortium. After 90 min of incubation with the bacterial consortium, the levels of **CTT** and **C-ProDOT** showed a decrease of more than 25% in comparison to the control sample, which consisted of surfactants incubated without bacteria in Lauryl tryptose broth (Figure 3).

### 3.5. Ecotoxicological Impact of Surfactants on Pure Microbial Strains

The short- and long-term effects of 100 mg/L of **CTT** and **C-ProDOT** on the growth of various bacterial strains were analyzed over a 24 h incubation period. The bacterial strains exhibited specific growth inhibition patterns induced by the surfactants, which varied based on whether the bacteria were Gram-positive or Gram-negative.

**Figure 4:**
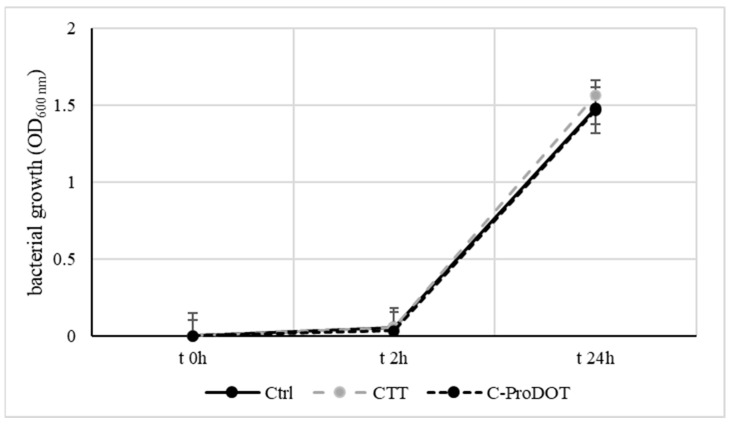
Gram-positive bacterial strain (*L. acidophilus*) growth exposed to 100 mg/L **CTT** and **C-ProDOT**. Control samples (Ctrl) were bacteria incubated without oligothiophene-based surfactants. All results are based on three independent experiments.

Neither the cationic-based terthiophene surfactant nor **C-ProDOT** surfactant showed significant growth inhibition of *L. acidophilus* (a Gram-positive bacterial strain) during the first 2 h of incubation compared to control samples (bacteria incubated without surfactants). Interestingly, a slight increase in the growth rate of *L. acidophilus* was noticed when exposed to **CTT** at 24 h (Figure 4).

**Figure 5:**
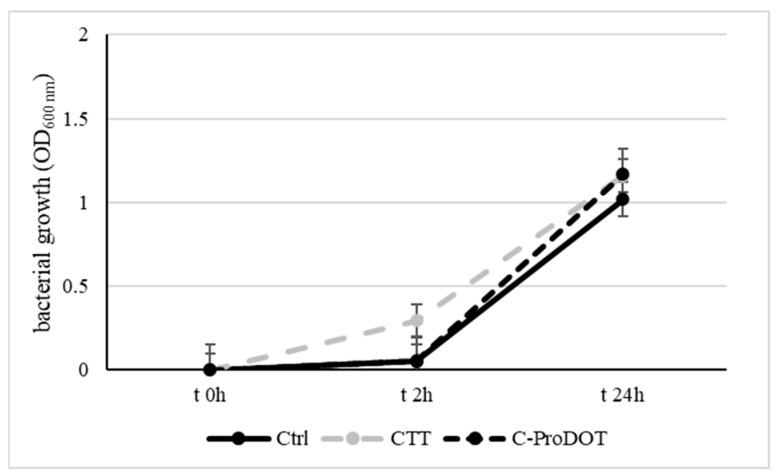
Gram-negative bacterial strain (*E. coli*) growth exposed to 100 mg/L **CTT** and **C-ProDOT**. Control samples (Ctrl) were bacteria incubated without oligothiophene-based surfactants. All results are based on three independent experiments.

For the Gram-negative bacterial strain *E. coli*, a slight increase in growth rate was observed after 2 h of incubation, with 100 mg/L of **CTT** (Figure 5). A similar slight increase in the growth rate of *E. coli* was observed at 24 h for both **CTT** and **C-ProDOT**.

**Figure 6:**
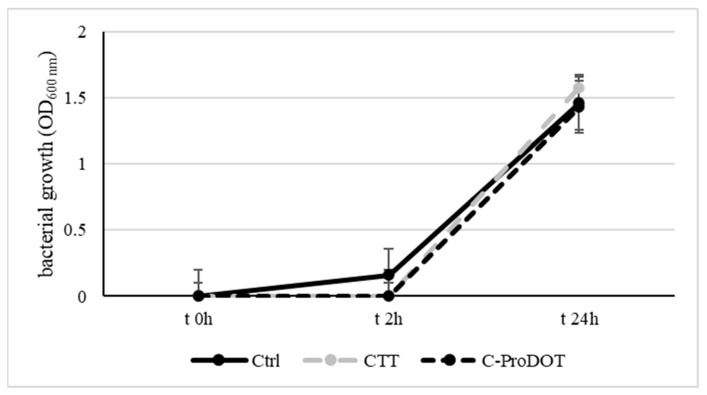
Gram-negative bacterial strain (*P. putida*) growth exposed to 100 mg/L **CTT** and **C-ProDOT**. Control samples (Ctrl) were bacteria incubated without oligothiophene-based surfactants. All results are based on three independent experiments.

In contrast, the Gram-negative bacterial strain *P. putida* reacted differently compared to *E. coli*. The growth of *P. putida* was inhibited by 100 mg/L of both **CTT** and **C-ProDOT** during the first 2 h of exposure. However, with longer exposure times to both surfactants, the inhibitory effect was reduced, and the bacteria showed a growth rate comparable to the control samples (Figure 6).

Most thiophene-based surfactants induced over 80% cell viability at concentrations up to 100 mg/L, although their effect varied depending on their specific chemical structure. This supports the biocompatibility of thiophene surfactants at low-to-moderate dosages. Studies related to structure–activity relationships have highlighted the importance of alkyl chain length and substituent groups in balancing the toxicity and efficacy of these surfactants [54].

### 3.6. Adhesion of Oligothiophene-Based Surfactants CTT and C-ProDOT with a Biological Host

**Figure 7:**
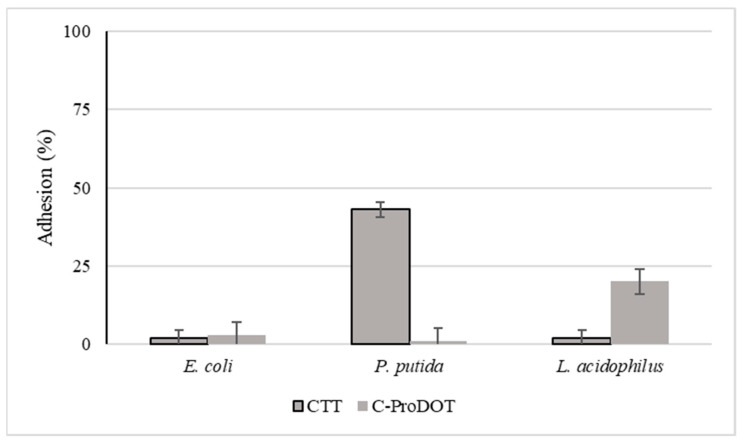
Adhesion of oligothiophene-based surfactants **CTT** and **C-ProDOT** on Gram-positive and Gram-negative bacterial strains. All results are based on three independent experiments.

The adhesion testing of **CTT** and **C-ProDOT** revealed a distinct pattern based on their structure. **C-ProDOT** demonstrated adhesion exclusively to Gram-positive bacterial strains (Figure 7). Approximately 20% of ProDOT-based ionic surfactant adhered to *L. acidophilus*-coated ELISA plates when compared to control samples, which were coated with Lauryl tryptose broth.

**Figure 8:**
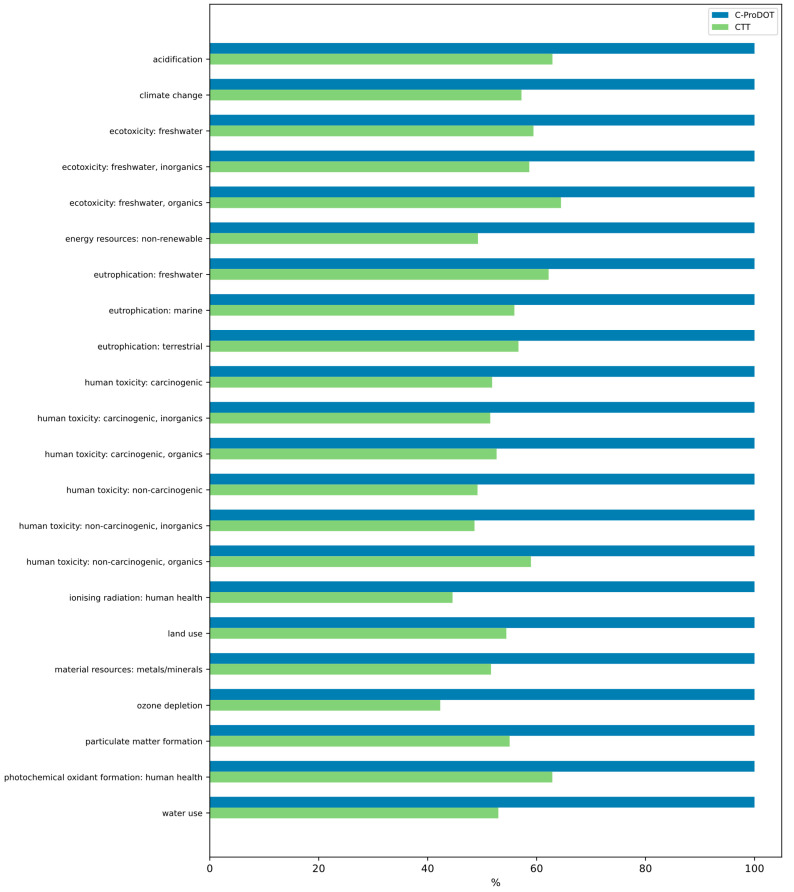
Characterization LCA results of cationic terthiophene (**CTT**) and ProDOT surfactants (**C-ProDOT**).

In contrast, **CTT** exhibited bacterial adhesion specifically to Gram-negative *P. putida*-coated ELISA plates and interacted with another Gram-negative bacterial strain, *E. coli* (Figure 8). Up to 50% of **CTT** was found to adhere to *P. putida* compared to the control samples.

The higher hydrophobic character of **CTT** compared to **C-ProDOT** appears to impart specific adhesion to Gram-negative bacteria, such as *P. putida*, but not on *E. coli*.

In contrast, **C-ProDOT** seemed to facilitate a specific interaction with *L. acidophilus*, a Gram-positive bacterium. The adhesion level of **C-ProDOT**, which contains two hydrophylic ethylene glycol side chains, was more than half the adhesion level of **CTT**, which possesses only one hydrophilic chain. This suggests that the presence of the extra hydrophylic side chain in **C-ProDOT** significantly enhances its adhesion properties.

### 3.7. LCA for Oligothiophene-Based Surfactants CTT and C-ProDOT Chemical Syntheses

LCA results based on **CTT** and **C-ProDOT** chemical syntheses provided a comprehensive understanding of their environmental impacts (Figure 8).

**C-ProDOT**-based ionic surfactants had a global warming potential (GWP100) of 17.352 kg CO_2_-equivalent per gram, significantly higher than CTT surfactants, with a GWP100 of 9.929 kg CO_2_-equivalent per gram.

The main reason for **C-ProDOT**’s higher GWP100 was the extensive energy used and reliance on organic solvents throughout its synthesis. Fossil-based energy accounted for 99.7% of **C-ProDOT**-based ionic surfactants’ GWP100, contributing 17.305 kg CO_2_-equivalent per gram. **CTT** surfactants followed a similar pattern, with 99.7% of their climate change impact stemming from fossil fuel emissions, equating to 9.904 kg CO_2_-equivalent per gram.

Energy use in non-renewable resources was another notable category. The synthesis of **C-ProDOT** required 1394.59 MJ-equivalent per gram, which was nearly double that of **CTT** surfactants, with 686.54 MJ-equivalent. This data showed greater energy demands for **C-ProDOT**, particularly due to a prolonged reaction time involving heating and stirring. In terms of human toxicity (carcinogenic), the ProDOT had an index of 1.61 × 10^−8^ comparative toxic units for humans (CTUh), which was 93% higher than **CTT** surfactants. with 8.34 × 10^−9^ CTUh. This was largely due to the use of dichloromethane and tetrahydrofuran, which known for their potential health risks. For **C-ProDOT**, a carcinogenic impact occurs primarily from organic solvents, which contribute over 80% to potential health risks.

The analysis of these LCA results highlights that **C-ProDOT** has an overall higher environmental burden than **CTT**, primarily due to increased energy demands and the nature of the chemicals used. **CTT**, while showing lower impacts, still requires attention due to its energy use and toxicity potential. In fact, both molecules showed significant environmental impacts in categories associated with fossil fuel usage, freshwater ecotoxicity and human toxicity. These results demonstrate the importance of adopting more sustainable practices, such as utilizing alternative, less harmful solvents and employing energy-efficient techniques in chemical synthesis. Therefore, implementing solvent recovery and recycling strategies can enhance sustainability and reduce waste. Moreover, transitioning laboratory operations to renewable energy sources would further reduce the climate change impact, particularly in processes where electricity contributes a significant share of the total environmental burden. Additionally, prioritizing the use of less toxic reagents could lower ecotoxicity and human health impacts, advancing the goals of green and sustainable chemistry.

## 4. Conclusions

New **CTT** and **C-ProDOT** surfactants were designed to have suitable optoelectronic properties. Indeed, the occurrence of potential π-stacking interactions during aggregation was shown to have a significant effect on photophysical properties, resulting in self-quenching. While this behavior was unfavorable for fluorescence, it could be advantageous for photocatalytic or other optoelectronic applications. The development of π-π stacks could facilitate the separation and migration of photogenerated charge carriers, effectively reducing recombination—a highly sought-after trait in electronic processes. The environmental impact of these newly designed surfactants’ chemical structures, which have potential for proton or electron transport, was mitigated due to their medium-to-high biodegradability. The growth of bacterial communities, which typically play a role in pollutant biodegradation during WWTP processes, was not adversely affected by these newly designed surfactants. Furthermore, the metabolic pathways of these bacteria appeared to biodegrade the newly designed surfactants, resulting in increased oxygen consumption.

There were no significant long-term toxic effects on specific Gram-positive or Gram-negative bacterial strains when these strains were incubated for 24 h in the presence of surfactants. Surfactants that exhibited no toxic effects on various bacterial strains became strong candidates for bacterial interactions and could serve as a foundation for the development of biosensor devices. **CTT** showed specific adhesion with Gram-negative bacteria, especially with *P. putida*, whereas **C-ProDOT** showed specificity for the Gram-positive bacterial strain *L. acidophilus*, exhibiting no interactions with Gram-negative bacterial strains such as *E. coli* and *P. putida*. Overall, the newly synthesized surfactants have the potential to transport protons or electrons, making them excellent candidates for biosensor assembly, especially given their lack of significant (eco)toxic effects on biological models based on various bacterial strains.

The LCA results show that **C-ProDOT** surfactants had greater environmental impacts compared to their **CTT** counterparts, particularly due to energy consumption and ecotoxicity. These issues could be addressed by adopting alternative solvents, enhancing energy efficiency and implementing renewable energy sources. These steps are essential to align synthesis practices with sustainability goals and reduce the ecological footprint.

## Figures and Tables

**Table 1:** BOD_5_/COD ratio as an indicator of biodegradability.

BOD_5_/COD Ratio	Biodegradability Classification
**<0.2**	non-biodegradable
**0.2–0.5**	biodegradable
**>0.5**	highly biodegradable

**Table 2:** Summary of the main spectroscopic and photophysical data for **CTT** and **C-ProDOT**.

Compound	THF				Water			
	λ_max_ (nm)	ε (L.mol^−1^.cm^−1^)	λ_em_ (nm)	ϕ (%)	λ_max_ (nm)	ε (L.mol^−1^.cm^−1^)	λ_em_ (nm)	ϕ (%)
**CTT**	354	8000	439	4.4 ± 0.2	364	10,000	453	<1
**C-ProDOT**	376, 392	8000, 6000	438	4.1 ± 0.1	380, 398	8000, 6200	480	<1

**Table 3:** COD and BOD_5_ values of cationic-based terthiophene surfactant and ProDOT-based ionic surfactant.

**Compounds/Parameters**	**COD**	**BOD_5_**	**BOD_5_/COD**
	mg O_2_/L	mg O_2_/L	
**CTT**	2594	841	0.32
**C-ProDOT**	2693	874	0.32

## Data Availability

The original contributions presented in this study are included in the article/Supplementary Materials. Further inquiries can be directed to the corresponding author.

## Supplementary Materials

The following supporting information can be downloaded at https://www.mdpi.com/article/10.3390/ma18184349/s1, Figure S1. ^1^H NMR spectrum (400 MHz, CDCl_3_) of **2**; Figure S2. ^13^C{^1^H} NMR spectrum (101 MHz, CDCl_3_) of **2**; Figure S3. High resolution ESI (positive mode) mass spectrum of **2**; Figure S4. ^1^H NMR spectrum (400 MHz, CDCl_3_) of **3**; Figure S5. ^13^C{^1^H} NMR spectrum (126 MHz, CDCl_3_) of **3**; Figure S6. High resolution ESI mass spectrum (positive mode) of **3**; Figure S7. ^1^H NMR spectrum (400 MHz, CDCl_3_) of **CTT**; Figure S8. ^13^C{^1^H} NMR spectrum (101 MHz, CDCl_3_) of **CTT**; Figure S9. High resolution ESI mass spectrum (positive mode) of **CTT**; Figure S10. ^1^H NMR spectrum (400 MHz, CDCl_3_) of **5**; Figure S11. ^13^C{^1^H} NMR spectrum (126 MHz, CDCl_3_) of **5**; Figure S12. High resolution ESI (positive mode) mass spectrum of **5**; Figure S13. ^1^H NMR spectrum (400 MHz, CDCl_3_) of **6**; Figure S14. ^13^C{^1^H} NMR spectrum (101 MHz, CDCl_3_) of **6**; Figure S15. High resolution ESI mass spectrum (positive mode) of **6**; Figure S16. ^1^H NMR spectrum (400 MHz, CDCl_3_) of **7**; Figure S17. ^13^C{^1^H} NMR spectrum (126 MHz, CDCl_3_) of **7**; Figure S18. High resolution ESI mass spectrum (positive mode) of **7**; Figure S19. ^1^H NMR spectrum (400 MHz, CDCl_3_) of **C-ProDOT**; Figure S20. ^13^C{^1^H} NMR spectrum (101 MHz, CDCl_3_) of **C-ProDOT**; Figure S21. High resolution ESI mass spectrum (positive mode) of **C-ProDOT**; Figure S22. Plot of the intensity of scattered light (in kilo counts per second) obtained for various concentrations of **CTT** prepared in deionized water. The intersection of the two lines in the intensity data corresponds to the critical micelle concentration; Figure S23. Plot of the intensity of scattered light (in kilo counts per second) obtained for various concentrations of **C-ProDOT** prepared in deionized water. The intersection of the two lines in the intensity data corresponds to the critical micelle concentration.
