# Supplementary material for: Sustainable Design and Environmental Effects of π-Conjugated Thiophene Surfactants for Optoelectronic Applications
Source: Materials (Basel). 2025 Sep 17;18(18):4349. doi: 10.3390/ma18184349 (PMC12471932; doi:10.3390/ma18184349)
Supplement: Supplementary file 1 [file materials-18-04349-s001.zip › materials-3830683-supplementary.pdf]

materials

MDPI

Supplementary material

Table of Contents

I. Characterization S1-S21
II. CMC Measurements S22-23

I. Characterization

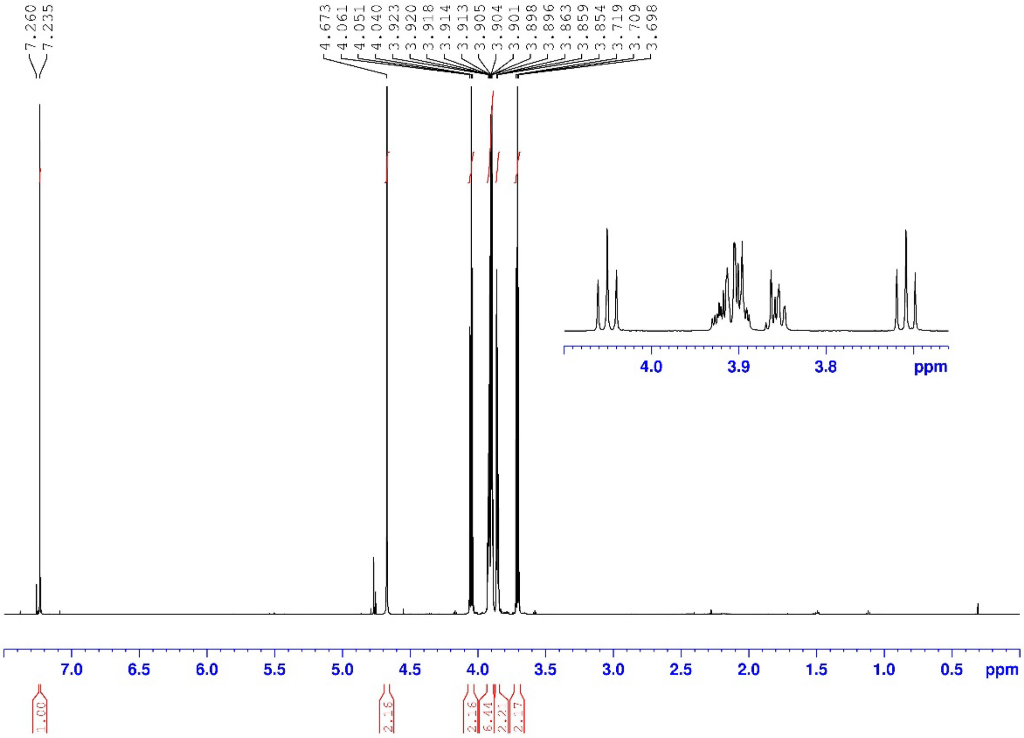

Figure S1. ^{1}H NMR spectrum (400 MHz, $CDCl_3$) of **2**.

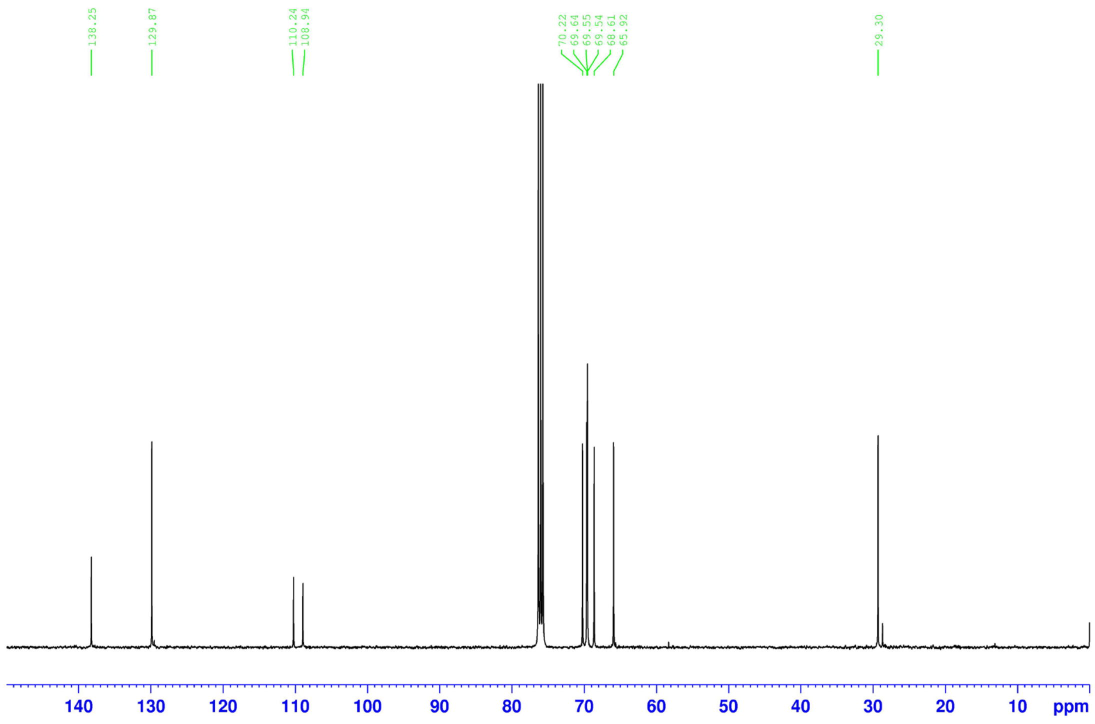

Figure S2. $^{13}C\{^{1}H\}$ NMR spectrum (101 MHz, $CDCl_3$) of **2**.

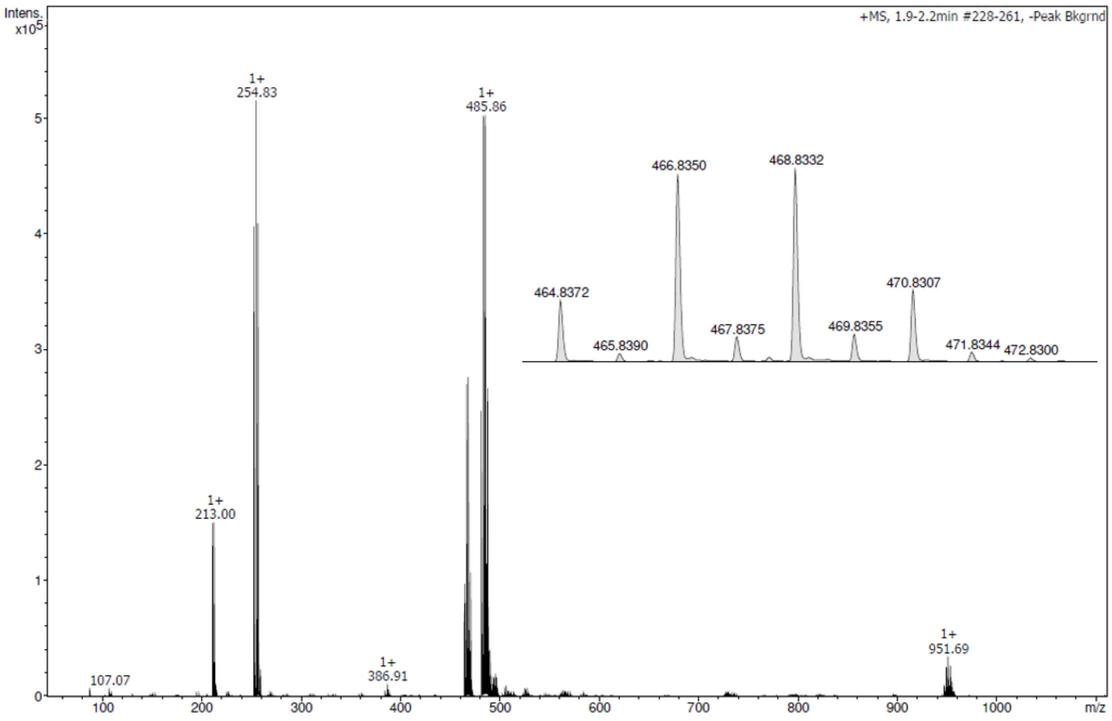

Figure S3. High resolution ESI (positive mode) mass spectrum of **2**.

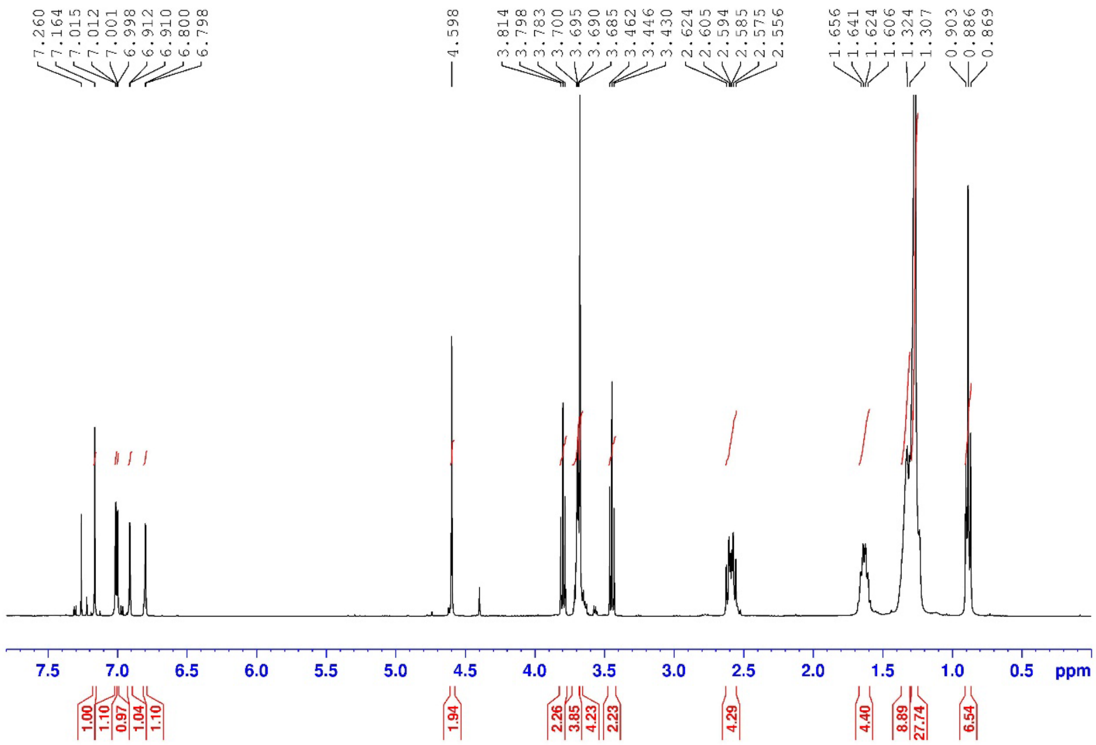

Figure S4. ^{1}H NMR spectrum (400 MHz, $CDCl_3$) of **3.**

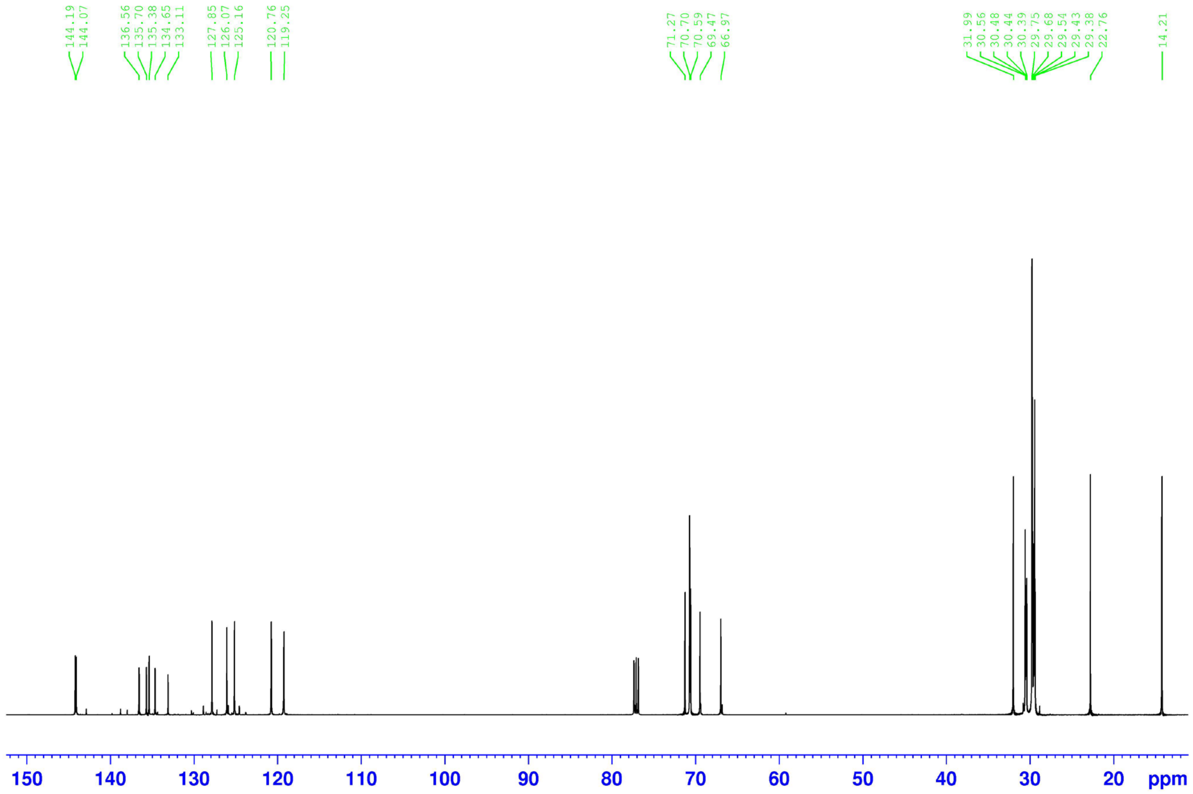

Figure S5. ^{13}C{^{1}H} NMR spectrum (126 MHz, $CDCl_3$) of **3.**

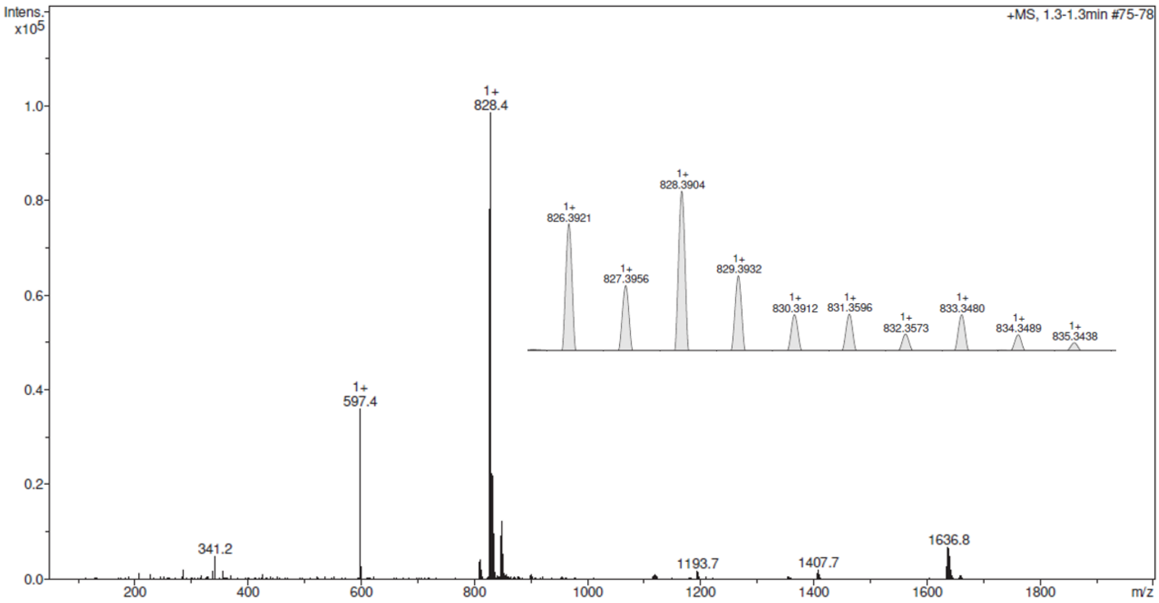

Figure S6. High resolution ESI mass spectrum (positive mode) of **3.**

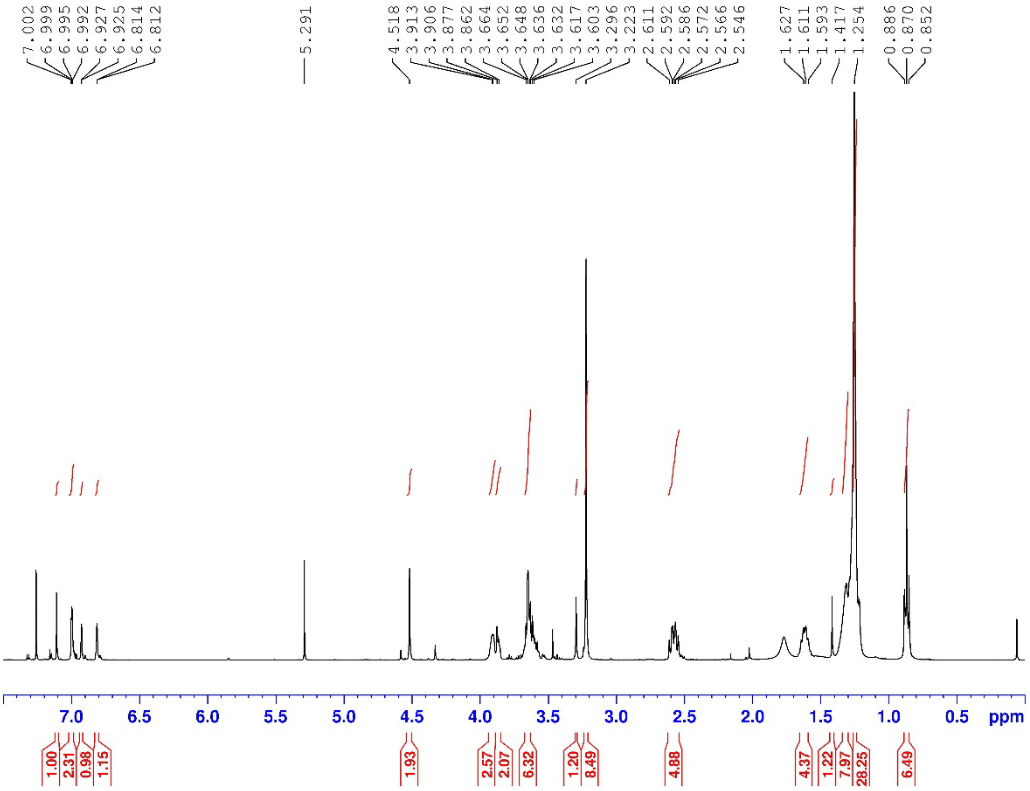

Figure S7. ^{1}H NMR spectrum (400 MHz, $CDCl_3$) of **CTT.**

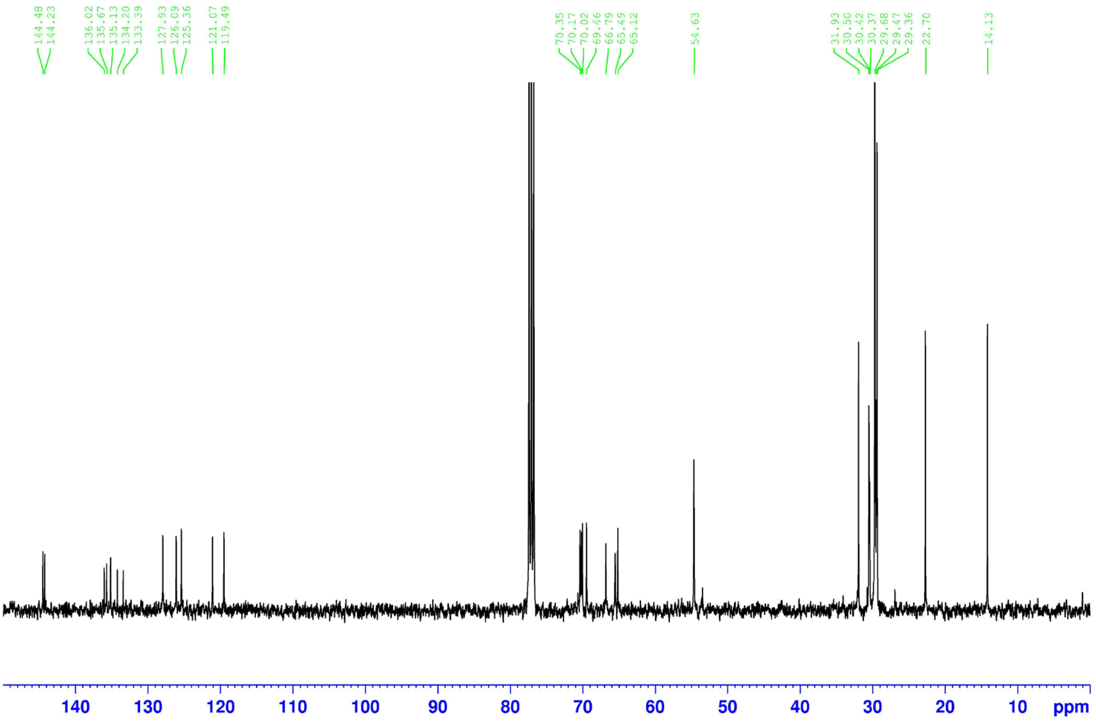

Figure S8. $^{13}C\{^{1}H\}$ NMR spectrum (101 MHz, $CDCl_3$) of **CTT.**

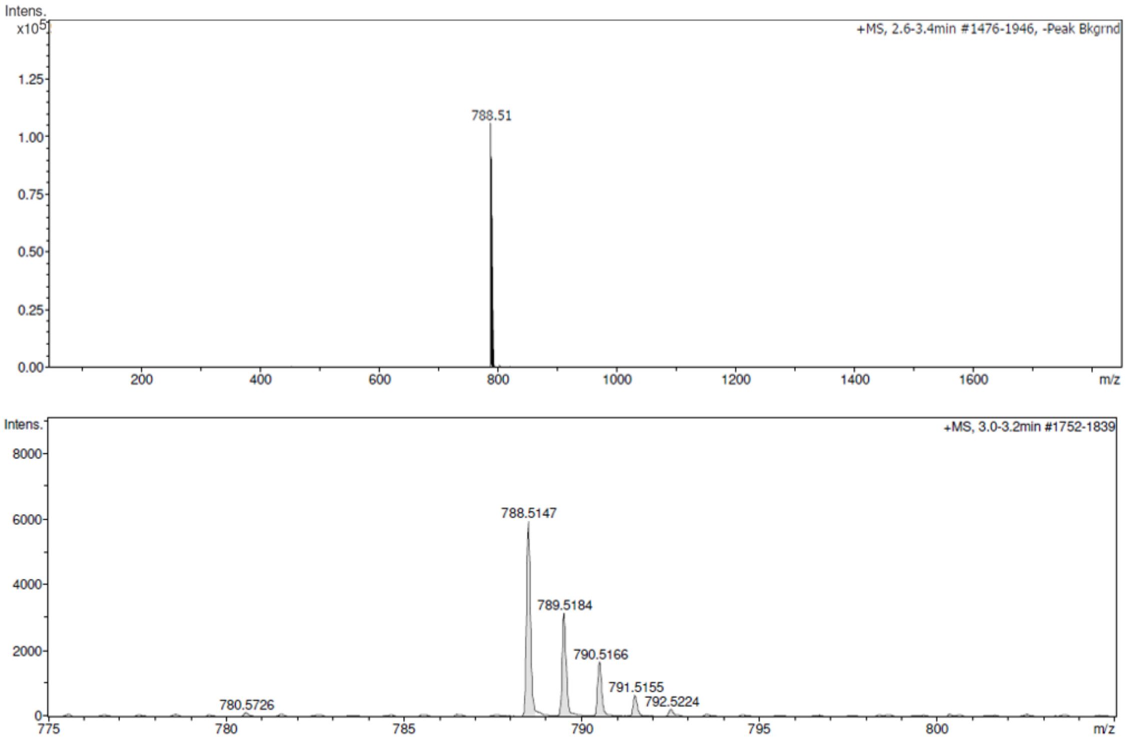

Figure S9. High resolution ESI mass spectrum (positive mode) of **CTT.**

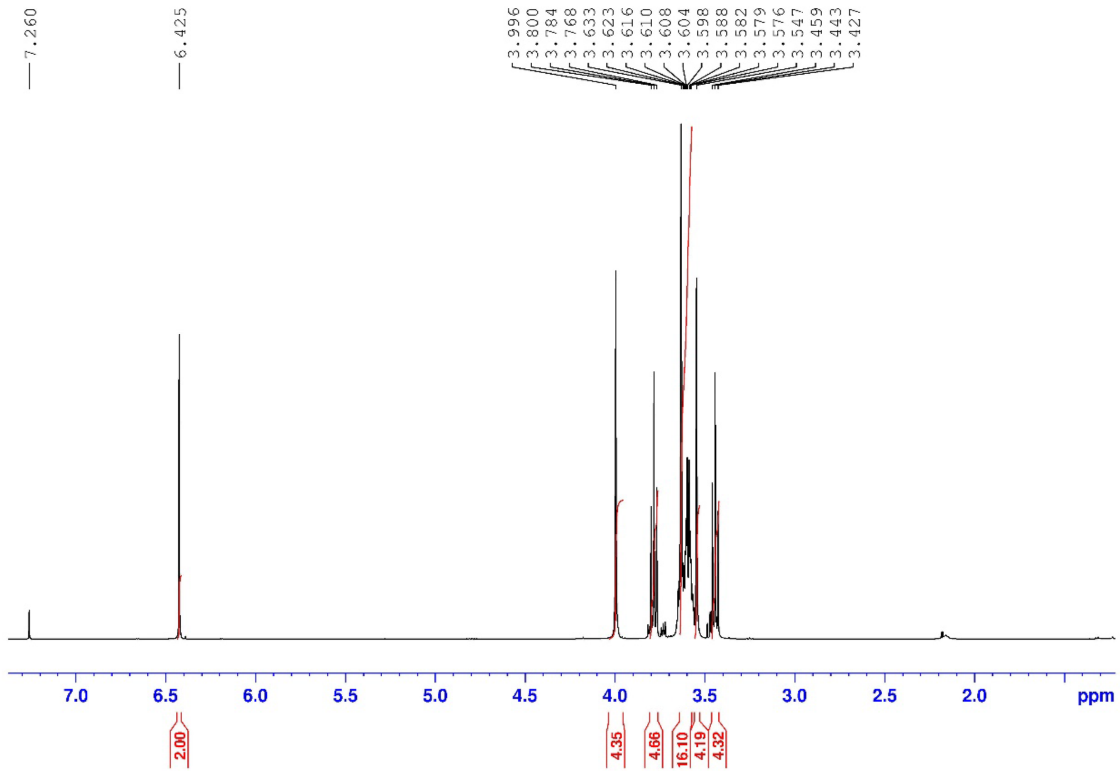

Figure S10. ^{1}H NMR spectrum (400 MHz, $CDCl_3$) of **5.**

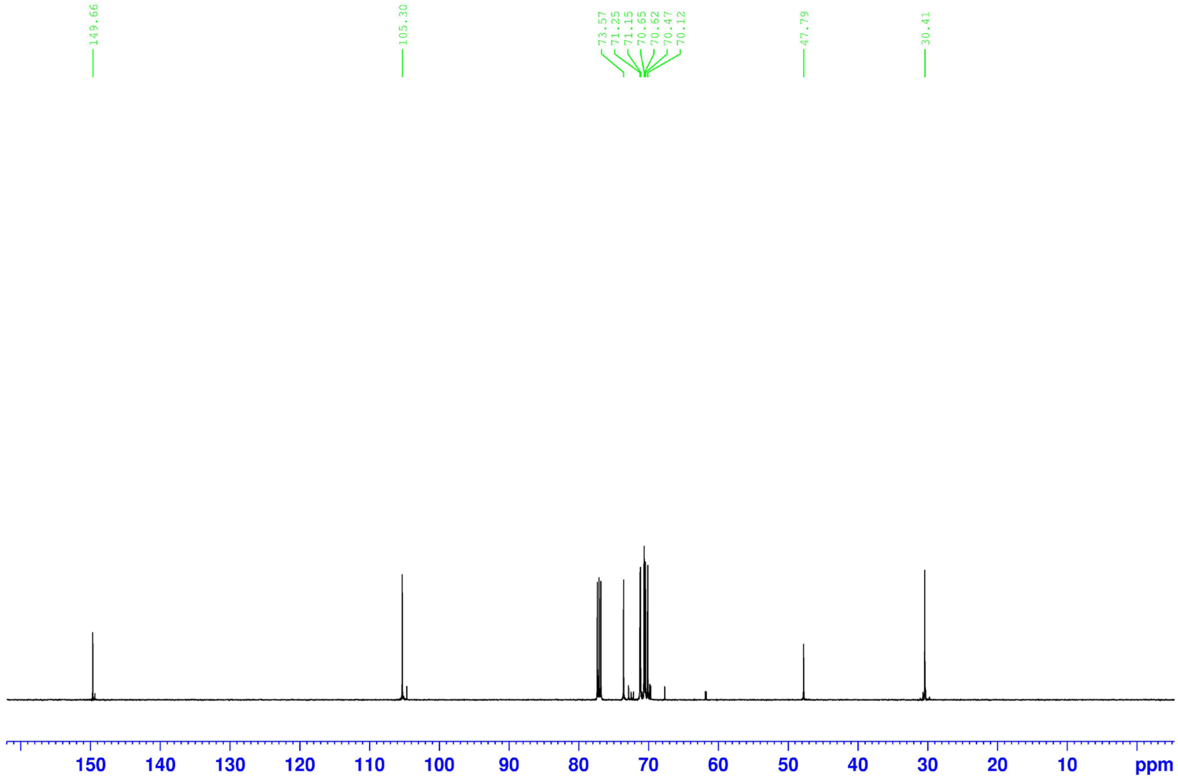

Figure S11. $^{13}C\{^{1}H\}$ NMR spectrum (126 MHz, $CDCl_3$) of **5**.

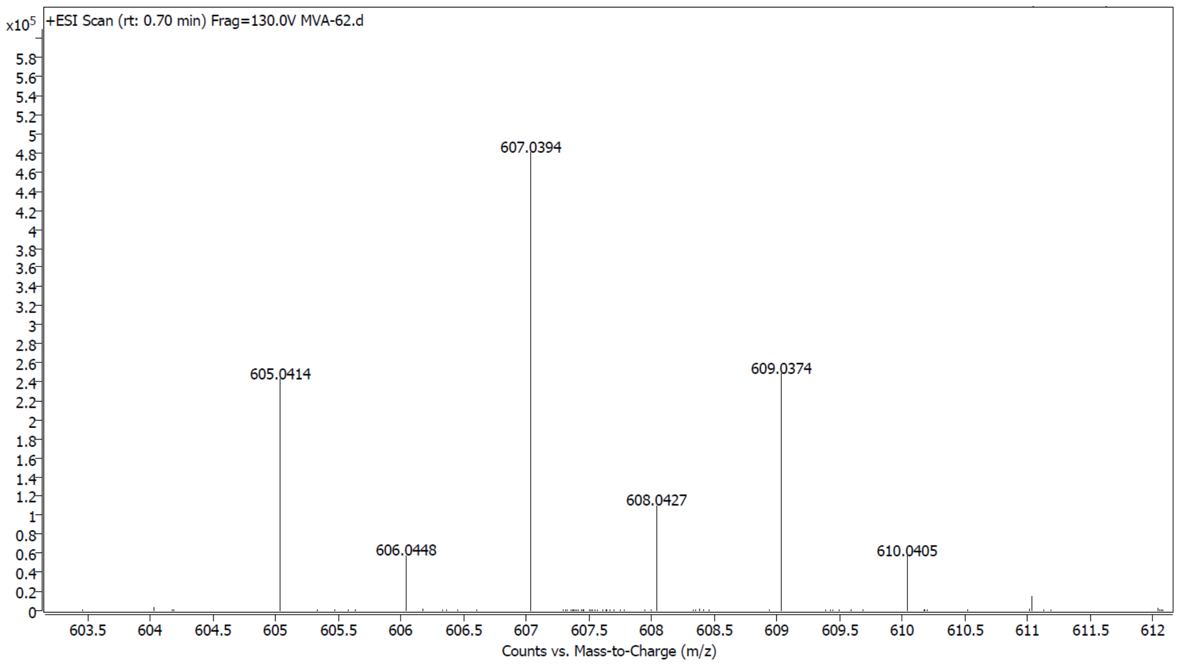

Figure S12. High resolution ESI (positive mode) mass spectrum of **5**.

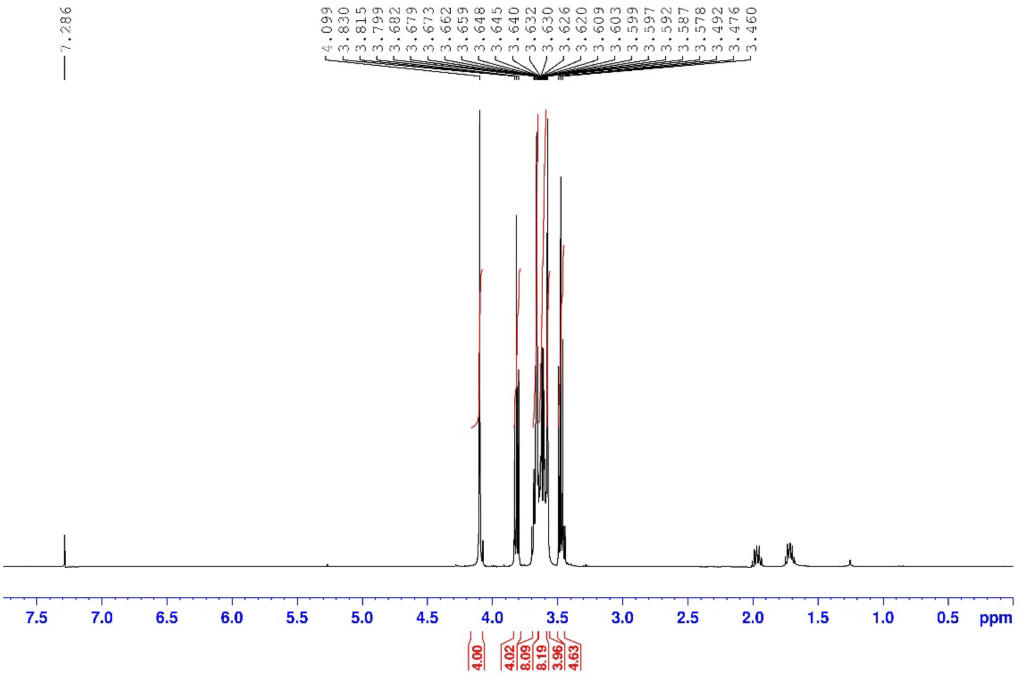

Figure S13. ^{1}H NMR spectrum (400 MHz, $CDCl_3$) of **6.**

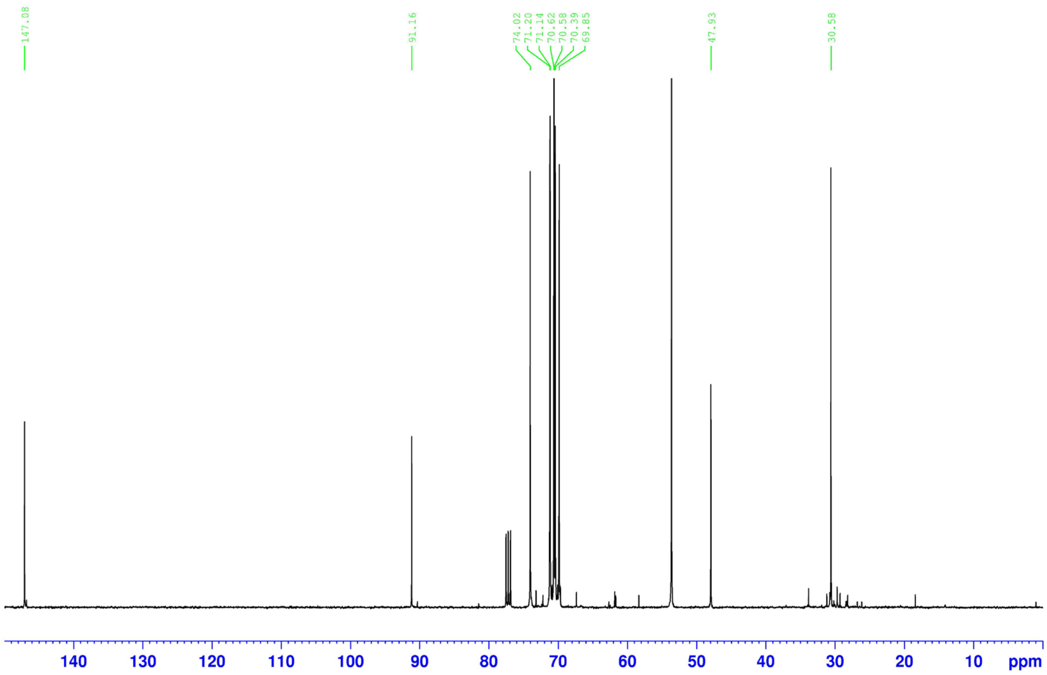

Figure S14. $^{13}C\{^{1}H\}$ NMR spectrum (101 MHz, $CDCl_3$) of **6.**

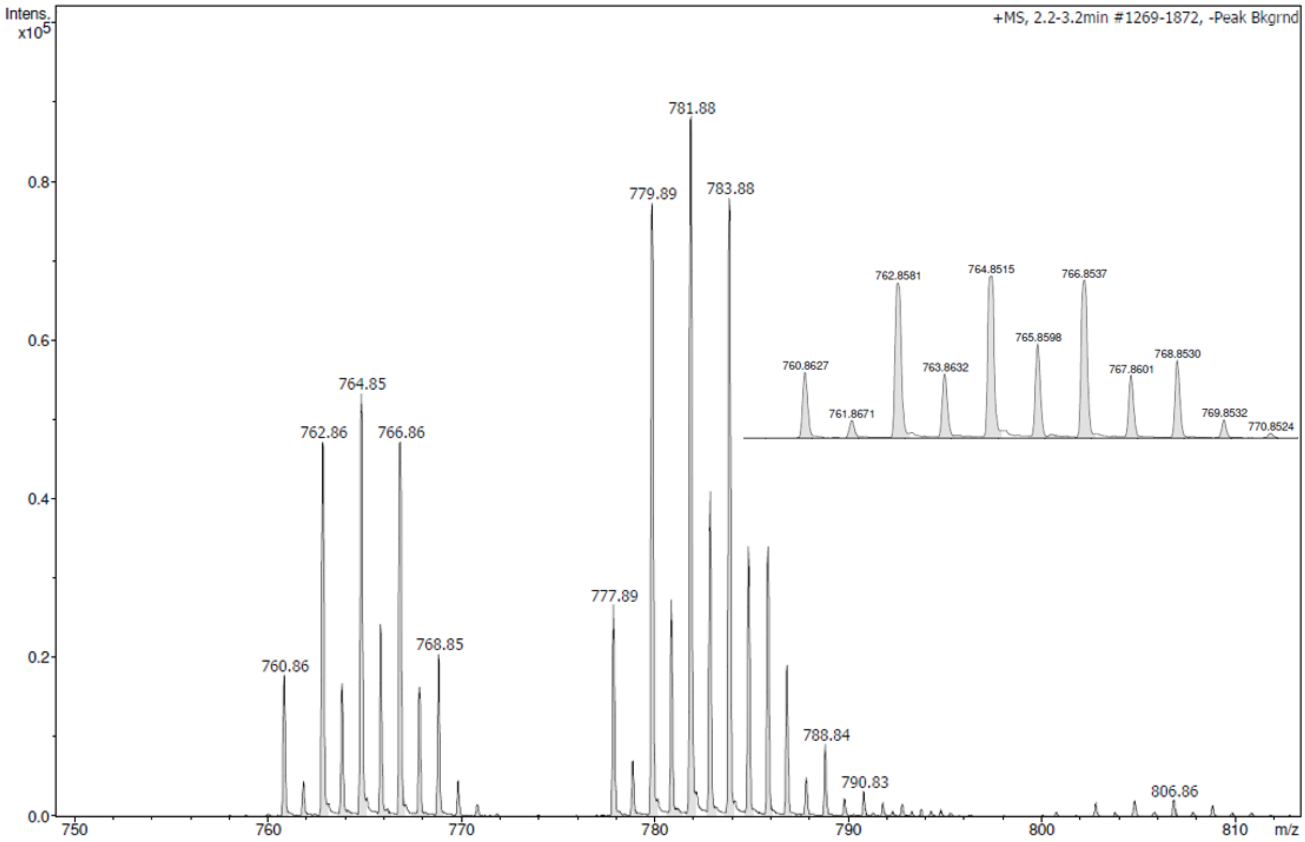

Figure S15. High resolution ESI mass spectrum (positive mode) of **6.**

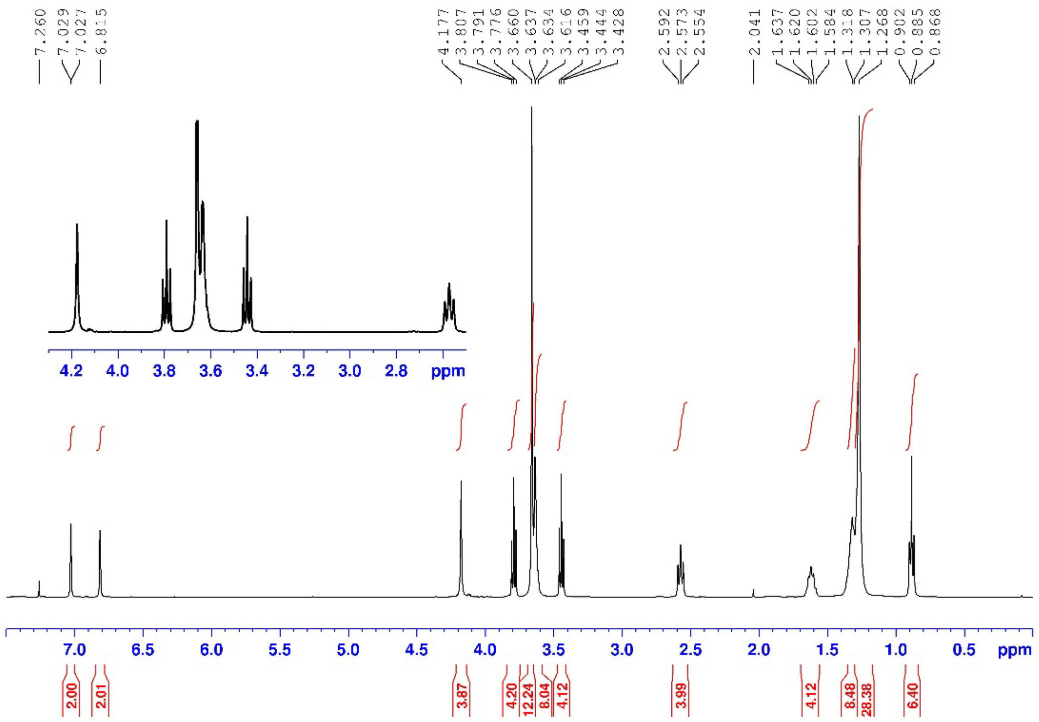

Figure S16. ^{1}H NMR spectrum (400 MHz, $CDCl_3$) of **7.**

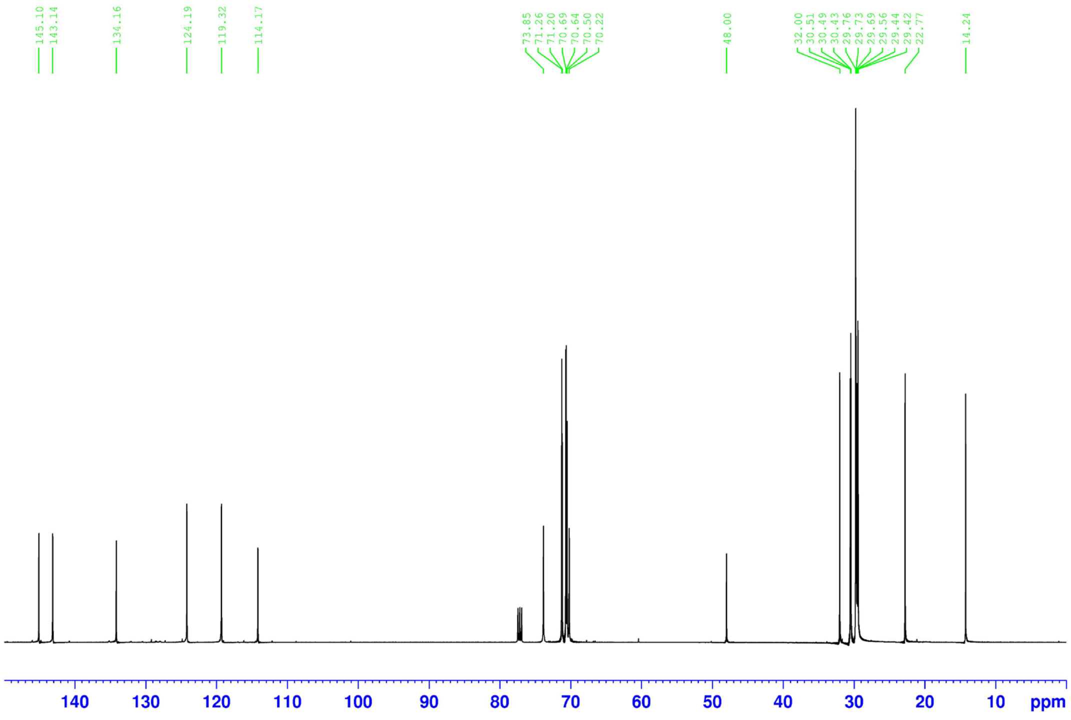

Figure S17. $^{13}C\{^{1}H\}$ NMR spectrum (126 MHz, $CDCl_3$) of **7.**

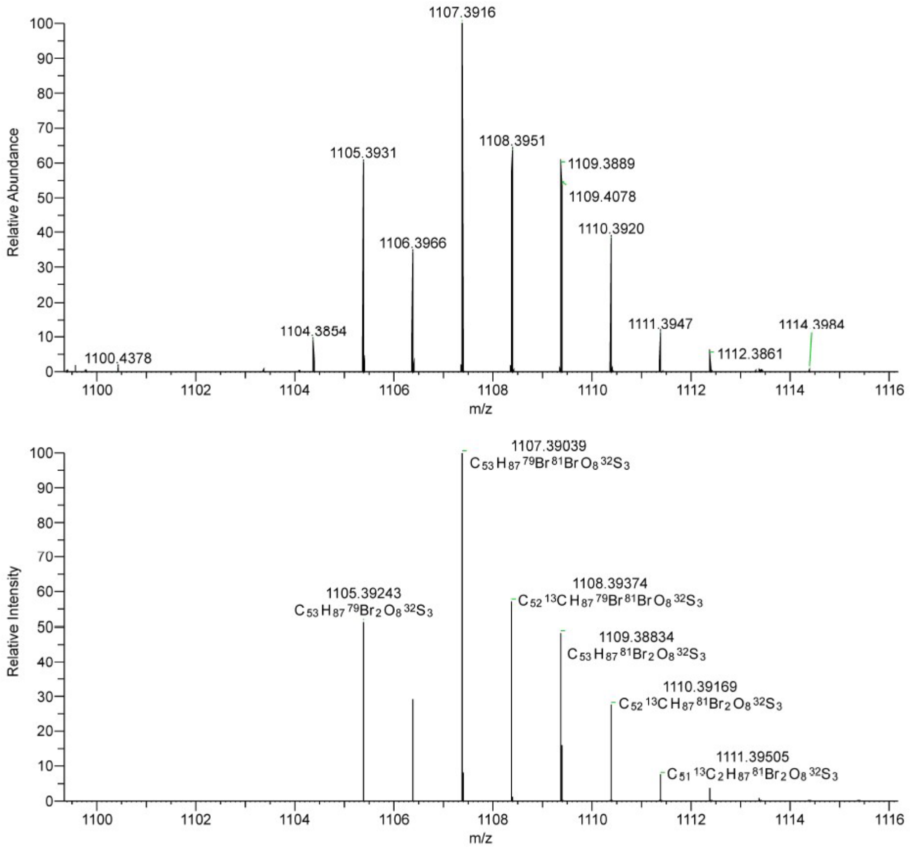

Figure S18. High resolution ESI mass spectrum (positive mode) of **7.**

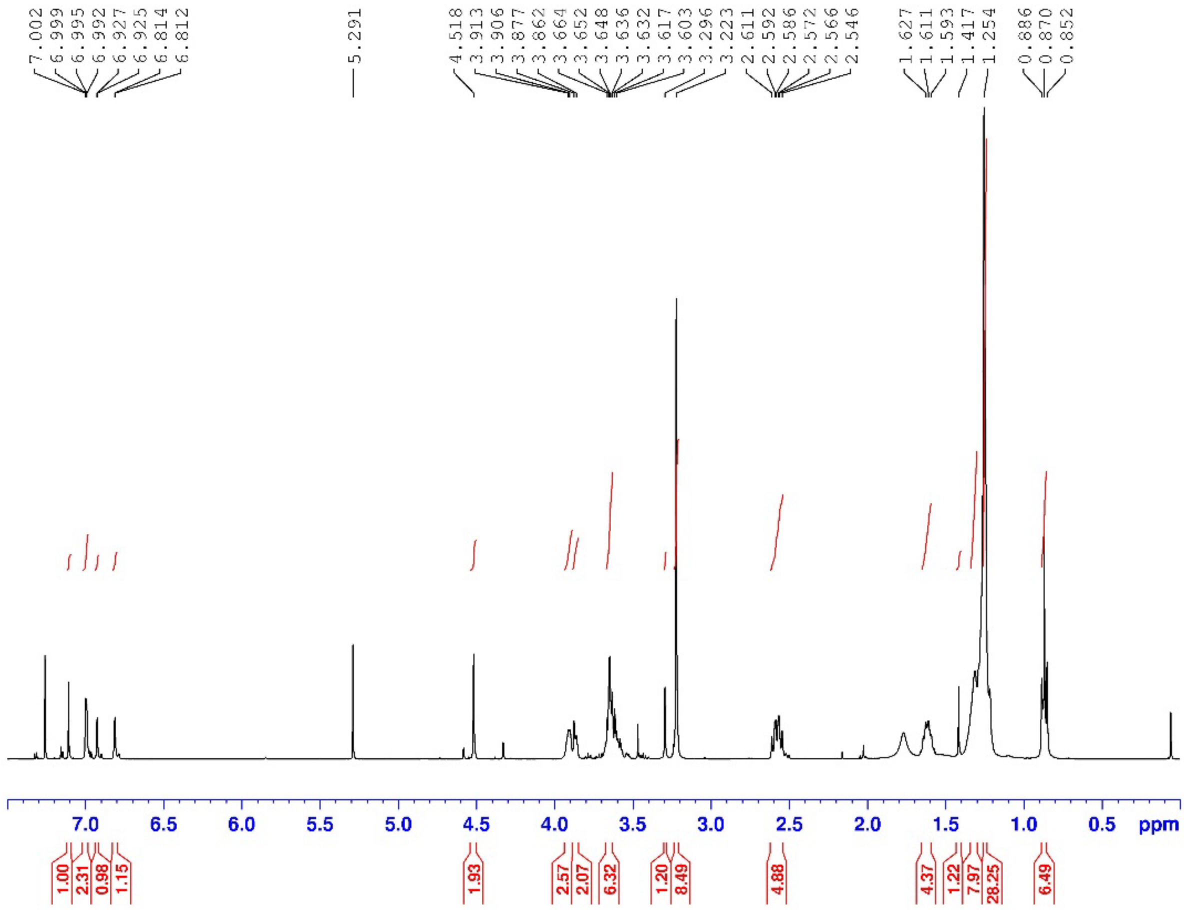

Figure S19. ^{1}H NMR spectrum (400 MHz, $CDCl_3$) of **C-ProDOT.**

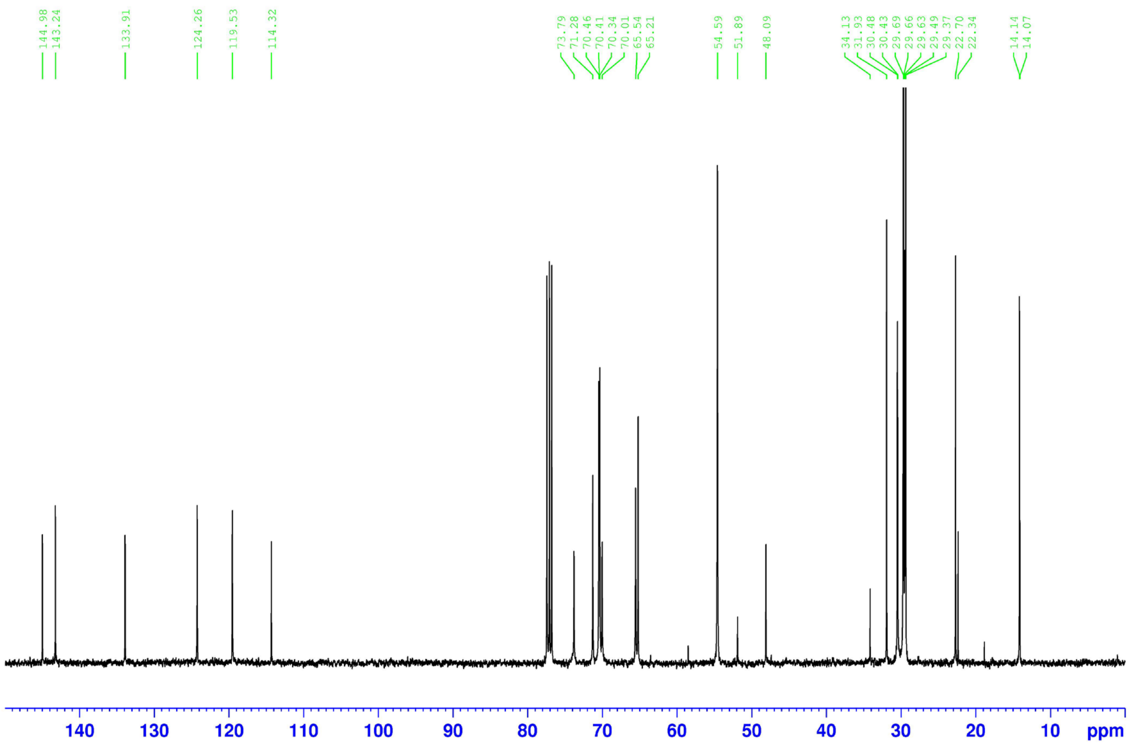

Figure S20. $^{13}C\{^1H\}$ NMR spectrum (101 MHz, $CDCl_3$) of **C-ProDOT.**

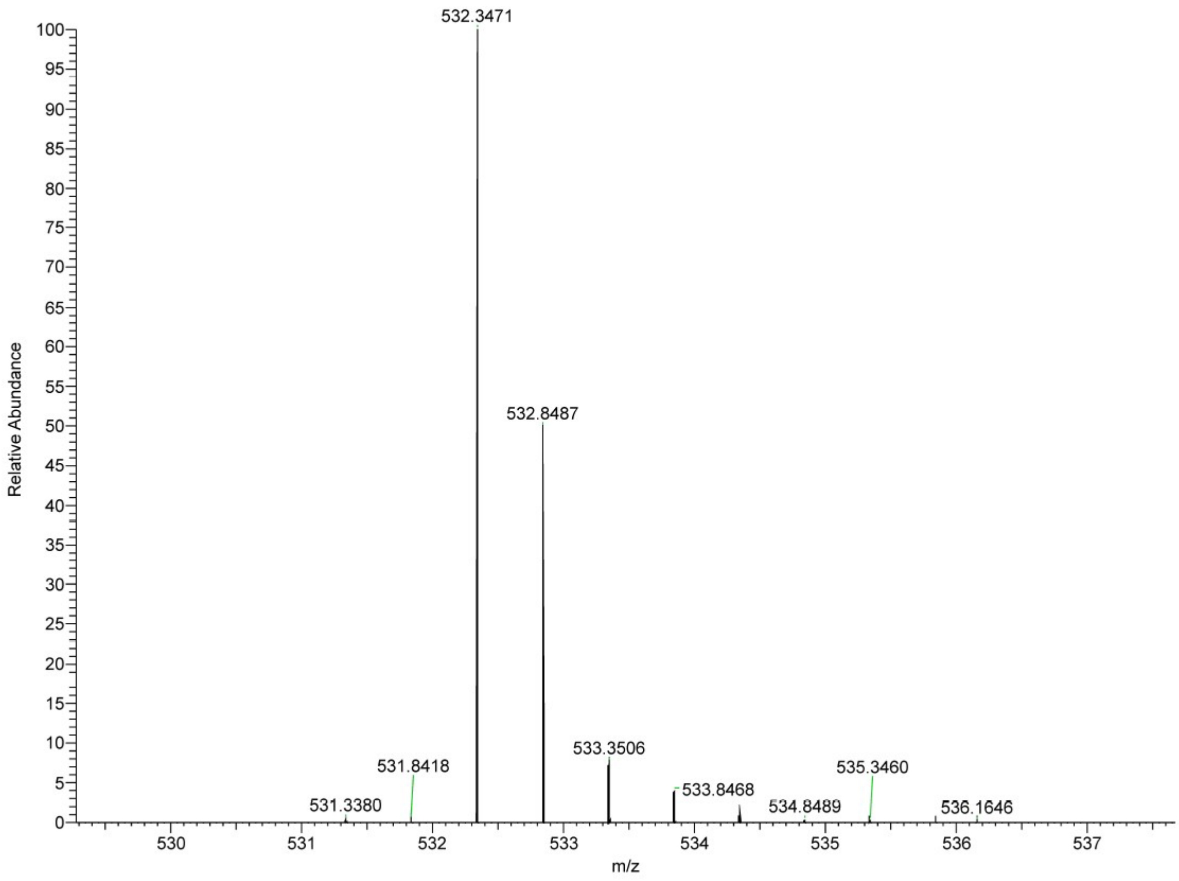

Figure S21. High resolution ESI mass spectrum (positive mode) of **C-ProDOT.**

II. CMC Measurements

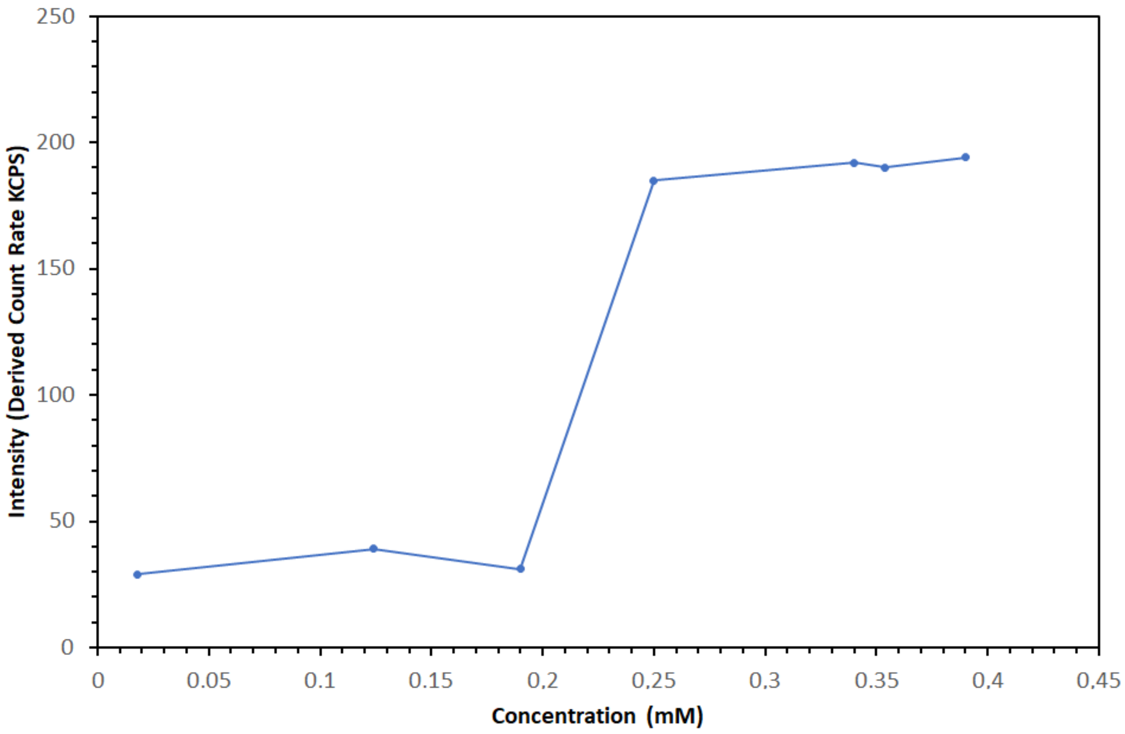

Figure S22. Plot of the intensity of scattered light (in kilo counts per second) obtained for various concentrations of **CTT** prepared in deionized water. The intersection of the two lines in the intensity data corresponds to the critical micelle concentration.

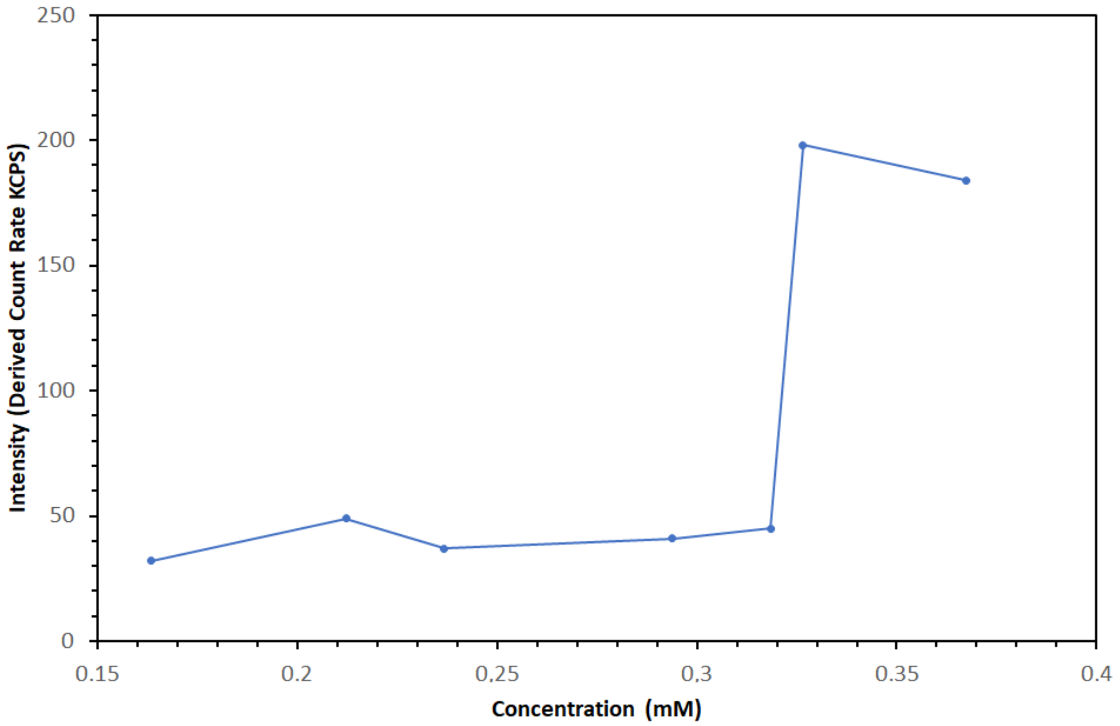

Figure S23. Plot of the intensity of scattered light (in kilo counts per second) obtained for various concentrations of **C-ProDOT** prepared in deionized water. The intersection of the two lines in the intensity data corresponds to the critical micelle concentration.
